# Robust operation of renewable virtual power plant in intelligent distribution system considering active and reactive ancillary services markets

**DOI:** 10.1038/s41598-025-06501-z

**Published:** 2025-07-02

**Authors:** Mohabbat Vafa, Mohammad Hossain Ershadi, Behdad Arandian

**Affiliations:** 1 Department of Electrical Engineering, Khomeinishahr Branch, Islamic Azad University, Isfahan, Iran; 2 Department of Electrical Engineering, Dolatabad Branch, Islamic Azad University, Isfahan, Iran

**Keywords:** Virtual power plant coupled with electric inverter, Smart distribution network, Energy market, Active and reactive ancillary services market, Adaptive robust optimization, Electrical and electronic engineering, Energy grids and networks, Power distribution

## Abstract

A virtual power plant consists of various sources, storage devices, and responsive loads. The operator of this unit can operate it as an energy storage device and transmitter in power distribution networks by controlling the active power of the aforementioned elements. This virtual unit is also connected to the grid with an electrical inverter, which can control active and reactive power between the grid and the virtual unit. Therefore, the system operator can gain financial benefits from different markets for sources, storage devices, and responsive loads. This study presents the operation of an intelligent distribution system (IDN) as a coupling of the virtual power plant and electric inverter (CVE). CVEs participate in energy and active (flexibility market) and reactive (reactive power market) service markets simultaneously. The deterministic formulation of the proposed scheme is responsible for maximizing the profits of CVEs in the markets for reactive power and energy. In this case, the problem is limited by the AC optimal power flow equations in the network and the operation model of CVEs. A nonlinear formulation and a linear approximation model are used in this scheme to achieve the optimal solution. An adaptive robust optimization (ARO) approach is applied to model uncertainties in energy prices, renewable energy, and mobile storage device energy consumption. Since flexibility modeling requires at least two uncertainty scenarios, the formulation of CVE participation in the flexibility market is further modeled. The CVE’s objective function in this scheme is to maximize profit in each of the markets listed above, and the model is constrained by the resulting robust model and the formulation of CVE flexibility. Finally, CVEs can improve network functionality and allow access to significant profits in these markets for power sources, storage devices, and responsive loads, as demonstrated by numerical results obtained from implementing this scheme on an IEEE 69-bus IDN. The proposed design can be applied in consumption areas such as industrial, agricultural, and residential sectors, leading to increased energy efficiency.

## Introduction

### Motivation

Since renewable energy sources (RES) produce clean electricity with very low pollution levels, wind and solar systems are increasingly gaining importance in modern power grids, particularly power distribution networks^1^. However, intensifying the availability of these resources along with the advantages of clean energy production with very low operating costs in power distribution networks will deteriorate the status of system operation and flexibility indices. Voltage profiles, network losses, and congestion on distribution lines are some operation indices to be considered. As more RESs are employed in a network, the risk of overvoltage, network losses, and congestion of distribution lines increases^1^. The concept of flexibility is defined as “*modifying the generation injection and/or consumption pathways in a reaction to an external price or activation signal to procure a service in an electrical system*”^2^. Thus, reducing the flexibility of a network makes the results of its energy utilization in real-time (RT) operation inconsistent with those of its energy forecast in day-ahead (DA) operation; hence, it is necessary to pay a fee or a penalty for imbalance in the energy market^2^. This is associated with the lack of control and management of the energy generated by RESs because consumers tend to consume low-cost energy, and network management tends to utilize less polluting resources. These sources inject their energy generation capacity into the network, which is proportional to the weather conditions and is uncertain^1^. As a result, to compensate for this issue, controllable resources are needed along with RESs.

Batteries and demand response programs (DRPs) are generally suitable choices due to very low time constants^3^. These elements are flexible sources that can compensate for the power swings of RESs in RT operation versus DA operation. The presence of these resources can improve network operation indices. It should, however, be noted that power systems or distribution networks must properly manage these resources to achieve these goals. To properly manage energy on a network, it is first necessary to utilize an aggregator framework, such as a virtual power plant (VPP), storage devices, and responsive loads^4^. Note that by establishing bilateral coordination between these elements and the operator, VPP can achieve a suitable operation and schedule for renewable and flexible sources. The optimal operation of a power distribution network can be achieved by coordinating the VPP operator and the distribution system operator (DSO). It should be noted that electric inverters or springs (ESs) have a bidirectional DC-AC converter that can control their load on the AC side^5^. It can regulate voltage as well as active and reactive power. In this context, the coupling of VPP and ES (CVE) is expected to play a key role in the integrated management of both active and reactive power. This will, in turn, improve various technical and economic indices. Furthermore, VPPs can benefit from the system’s participation in different energy and ancillary service markets.

### Literature review

Research has been conducted on the operation of power sources in various fields, storage systems, and responsive loads in intelligent distribution systems (IDNs). For example, Ref^6^. investigates IDN robustness during the aggregation of distributed generation and electric vehicles. A combination of these elements participates in the markets for reactive power and energy simultaneously. It is observed that the cost of charging electric vehicles (EVs) in the case of participating in the two mentioned markets is reduced by 30% compared to the case in which they don’t. The energy management (active power exchange) of power sources, storage, and responsive loads in the form of a microgrid (MG) connected to the power distribution network is presented in^7^, in which only the participation of MGs in the energy market is considered. Ref^8^. presents a multi-objective optimization for power sources and storage devices within MGs. Multi-objective optimization is used to simultaneously model the operation, reliability, and environmental indicators. This is the case for an unbalanced distribution network in^9^. Based on the results of^8,9^, it is observed that by optimally managing internal resources and storage, MG operators can simultaneously improve the status of several technical indicators in the network compared to the analyses of power flow while providing clean energy. The flexible operation of ES-coupled batteries in MGs with renewable sources is modeled in^10^. Based on the results, the battery-ES coupling system’s active and reactive power can be controlled simultaneously.

Several studies have been conducted on VPP operations. Reference^11^. presents a two-layer formulation for the presence of VPPs in an IDN. Its upper-level formulation states the model of participation of renewable resources and aggregation of EVs in the form of VPP in the energy and reserve markets. The operation of the IDN is mentioned at the lower level problem to reduce energy losses and voltage deviations. In^12^, the VPP format is used to coordinate responsive loads and wind turbines. Then, the involvement of this VPP in the DA and RT energy markets and the balance market is modeled. The work of^12^ is assessed in^13^. The only difference is that the VPP format is employed to coordinate the storage, wind turbine, and responsive loads. In^14^, it is hypothesized that VPP elements can simultaneously control active and reactive power and can contribute to the harmonic compensation of nonlinear loads. Accordingly, VPP can play a key role in improving operation indices and power quality. In^15^, the planning-operation model of VPPs in an IDN is studied. It shows that VPP can significantly improve the operation indices of power distribution networks through the proper sizing and siting of power sources and storage. It can have minimal planning costs. Ref^16^. examines the role of flexible VPPs such as photovoltaics (PV), hydropower, and pumped storage in the energy market. A study has been conducted on the operation of active distribution networks with VPPs^17^, in which the problem is bi-level. With the growing concern of global climate change in mind, Ref^18^. provides a novel data-driven approach to improve the operation of micro-grids based on a risk-averse flexi-intelligent energy management system (RFEMS). It takes into account the availability of diesel generators, EVs, ES, and RESs, as well as flexibility resources (FRs) with a DRP. A novel bi-level multi-objective model based on a flexible power management system is presented in Ref^19^. to maximize the flexibility of MGs. It takes into account the availability of flexible and renewable resources, such as energy storage systems, DRPs, and integrated electric inverter units with EV parking. In^20^, a hybrid distributed operation of EVs and RESs parking lot is proposed as a means of overcoming the obstacles caused by the intermittent nature of RESs and other uncertainties for building a reliable contemporary MG. The unscented transformation (UT) method-based stochastic programming is used to represent the uncertainties related to load, energy price, RESs, and availability of MG equipment in the context of a hybrid stochastic/robust optimization (HSRO) problem. To realize the robust potential of EVs in raising MG indices, the bounded uncertainty-based robust optimization (BURO) is used to predict the uncertain characteristics of an EV parking lot. A novel approach to managing the sporadic character of RESs and streamlining their integration into the smart active distribution network is presented in^21^. Ref^22^. reports a robust model for the energy storage system and demand-side management in the form of VPP. By analyzing random factors in the VPP operation, the robust model dynamically provided the operational constraints. Table 1 summarizes the research background.

**Table 1:** Classification of recent research works.

References	Considering the model of the electric inverter in VPP	Participation of VPP in markets	Model type for uncertainty parameters
^6^	No	Energy and reactive ancillary service	Adoptive robust programming
^7^	No	Energy	Scenario-based stochastic programming
^8^	No	–	
^9^	No	–	
^10^	No	–	
^11^	No	Energy and reserve regulation	
^12^	No	Energy	
^13^	No	Energy	
^14^	No	–	
^15^	No	–	
^16^	No	–	
^17^	No	–	
^18^	Considering the coupling of ES with EVs or storage units	–	
^19^		–	
^20^		–	Considering a hybrid stochastic and robust model
^21^		Energy	Scenario-based stochastic programming
^22^	No	–	Robust programming
Proposed scheme	Yes	Energy, active, and reactive ancillary services	Adaptive robust programming

### Research gaps

Table 1 compares the proposed scheme with the research background, highlighting the limitations of the research background and the innovations of the proposed scheme. According to this table, the main research gaps in the research background are: (1) insufficient attention to the combination of VPP and ES, although this system is capable of simultaneously controlling active and reactive power, (2) VPP’s participation in the energy market although it can have financial benefits in the ancillary services market, and (3) modeling uncertainties with stochastic optimization, although this technique increases the size of the problem and is unable to obtain a robust solution. Details of research gaps are as follows:

- In most studies, the model of energy (active power) exchange has been established in VPPs and their elements. However, some studies have considered a simultaneous exchange of power between the VPP and the network and its internal elements. In these conditions, a reactive power control element converter, such as a DC-AC converter, is used for each power source and storage. Note that within a CVE, internal components can only exchange energy among themselves. Nonetheless, ES, which is a DC-AC converter and has a controllable load on its AC side, controls the network’s reactive power exchange. In addition, by controlling the load on its AC side, it can regulate the voltage of the connection point to the network, which then produces an almost smooth voltage profile in the network. ES was proposed as a decentralized mechanism to bring voltage regulation and stability to the grid when installed at the customer’s end. This work focuses on the control aspect of the ES used for providing voltage regulation to the critical load by manipulating the voltage across the non-critical load, amidst fluctuating voltage inflicted by renewables, through injecting voltage with appropriate magnitude and phase^23^. However, ES and VPP coupling have rarely been addressed.
- Due to having non-renewable sources of energy, storage, and responsive loads, VPP can participate in both energy and ancillary service markets simultaneously and gain a suitable financial benefit for its elements. This has been studied more in the energy market, but less research has focused on the VPP participation model in reactive ancillary services markets or reactive power markets. Nonetheless, because the internal elements of VPP can control active power, VPP can play an effective role in enhancing network flexibility with renewable sources. It can participate in active ancillary services markets, such as flexibility markets, and benefit from them. This has been subject to limited research. Simultaneous modeling of their participation in energy and ancillary services markets has been dealt with by a few researchers.
- The operation problem generally has an execution step of less than one hour. The speed of solution convergence is of special importance in these problems^6,10^. In the problem of VPP operation, there are many uncertain parameters associated with load, energy price, renewable power, the energy consumption of mobile storage devices, and other items. Stochastic programming has been used in most research to model these uncertainties. As a result, this method will reduce the speed of convergence since it requires a significant number of scenarios to produce a reliable solution. To compensate for this, robust modeling of uncertainties is appropriate because it has only one scenario, which is the worst-case scenario. The resulting optimal point will then lead to the extraction of an optimal solution that is robust to the prediction error of uncertain parameters.

### Contributions

Given the research gaps mentioned above and to compensate for these cases, this paper presents an analysis of the robustness of CVEs in IDN, in which CVEs simultaneously participate in energy and active and reactive ancillary service markets. The reactive and active ancillary service market includes a reactive power market and a flexibility market, respectively. In the deterministic formulation, this plan is responsible for maximizing the profits of CVEs in the energy and reactive power markets. It is constrained by the model of the best AC power flow in IDN in the presence of RESs, the operation model of power sources, storage, and responsive loads in a VPP format, and ES formulation. The proposed scheme has a nonlinear formulation for which the solutions obtained by different solvers are not the same. Its convergence time is long. The coefficient of confidence in its response is low^6,10^. However, this research presents a linear approximation model, whose solvers can find the best solution in the shortest time. In other words, there is generally less than 6 degrees of deviation between the sending and receiving buses of the distribution line in the distribution network^1,2^. A conventional piecewise linear linearization technique can be used to take such assumptions into account. In addition to voltage magnitude linearization, a suitable linear approximation model can be obtained for AC power flow in IDN, which has a low computational error^1^. The allowable capacity of distribution lines, substations, and ES generally has a circular plane. By substituting a regular polygon plane instead of a circular one, the distribution line permissible capacity can be approximated by a linear approximation with little computational error, substations, and ES^11^. A linear approximation model with low computational error can be used to formulate the proposed scheme. Next, the parameters of load, market price, renewable power, and energy consumption of mobile storage devices (EVs) are uncertain. ARO is used to model them in this method. Since the objective function of the proposed scheme is *max*, the worst-case scenario is obtained from the expression *min* in the objective function in this method. In the robust model of the proposed scheme, there is a *max*-*min* form. Then, using the duality theory, the objective function has the form of *min* or *max*^6^. Next, to model the flexibility market, it is necessary to calculate the flexibility of CVEs. In comparison with a deterministic model, worst-case scenarios differ greatly, equaling the active power of the flexibility source. Thus, in the new problem, the objective function is equal to maximizing the total profit of CVEs in energy, reactive power, and flexibility markets, and the problem constraints include the obtained constraints of the robust model and the flexibility model of CVEs. To improve the convergence speed, the paper suggests using the Benders decomposition (BD) algorithm.

Lastly, the research contributions, novelties, and strengths can be summarized as follows:

- Modeling the simultaneous participation of CVEs in energy and active (flexibility market) and reactive (reactive power market) ancillary service markets,
- Modeling the optimal operation of CVE in the IDN by considering the simultaneous management of reactive and active power and voltage regulation in the mentioned network,
- Simultaneously and robustly simulating load, market price, renewable power, and mobile storage devices’ energy consumption, and.
- Simultaneously modeling economic, operations, and flexibility indicators.

### Paper organization

The rest of the paper is organized as follows. A deterministic formulation of the proposed scheme is given in “IDN operation in the presence of CVE (deterministic model)” for both linear and nonlinear models. Then, “Robust flexible operation of CEVs in IDN” presents robust modeling of uncertainties based on ARO. The numerical results are analyzed in “Numerical results”. Finally, “Conclusion” presents the conclusions.

## IDN operation in the presence of CVE (deterministic model)

### The system description

**Fig. 1:**
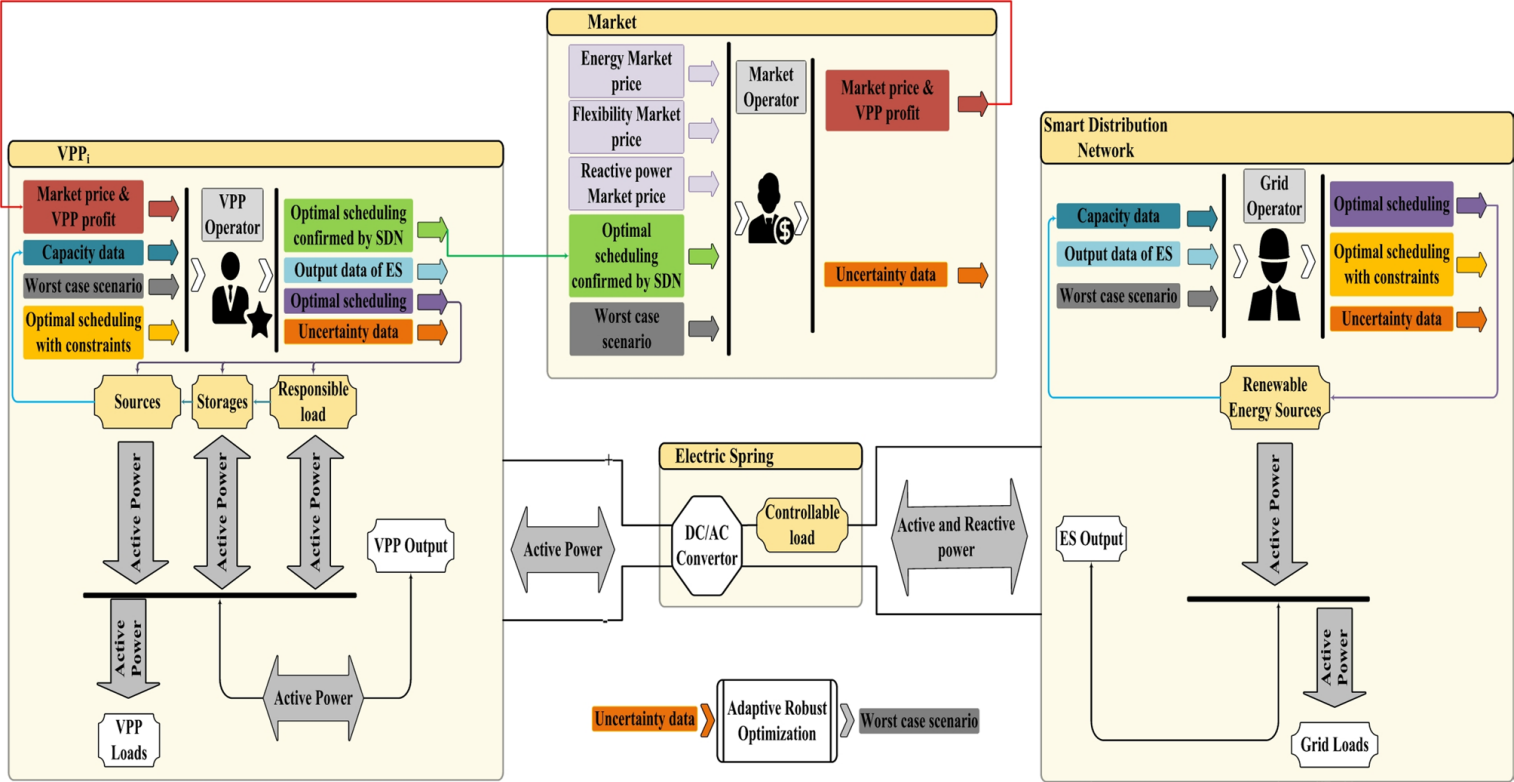
The framework of the proposed scheme.

According to Fig. 1, the VPP operator is in two-way coordination with the market operator and the distribution system operator. It manages the energy of resources, storage devices, and loads based on its own goals, as well as the market operator and network operator objectives. In other words, it maximizes the financial benefit gained from the energy and ancillary service market based on its own goals. It also controls its injected power into the network based on the network operator’s goals until the network operation limits are met. This condition is met when there is a smart platform in the network, VPP, and the market. Hence, the term “intelligent distribution network” was used in this article. Based on the proposed structure in Fig. 1, VPP can be owned by a private company, or it can be owned by the power distribution network.

### Original non-linear formulation

This section presents the deterministic formulation of IDN operation in the presence of CVEs. The plan is to maximize the profits of CEVs in the energy and reactive power markets. The IDN and CVE operation model must adhere to AC optimal power flow (AC-OPF) constraints. This plan is formulated as follows:

1 $$\hbox{max} \,\,\,\,\,\,F=\sum\limits_{{i,\tau }} {{\rho _{E\,\tau }}\left( {{P_{CVE\,i,\tau }}+{K_Q}{Q_{CVE\,i,\tau }}} \right)}$$

Subject to:

2 $$\begin{gathered} {P_{DS\,b,\tau }}+{P_{PV\,b,\tau }}+{P_{WT\,b,\tau }}+\sum\limits_{i} {{A_{i,b}}{P_{CVE\,i,\tau }}} - \sum\limits_{k} {{B_{b,k}}{P_{DL\,b,k,\tau }}} =\, \hfill \\ \,\,\,\,\,\,\,\,\,\,\,\,\,\,\,\,\,\,\,\,\,\,\,\,\,\,\,\,\,\,\,\,\,\,\,\,\,\,\,\,\,\left( {1 - {C_b}} \right){P_{L\,b,\tau }}+{C_b}{P_{L\,b,\tau }}\left( {Z{{\left( {\frac{{{V_{b,\tau }}}}{{V_{{b,\tau }}^{{LF}}}}} \right)}^2}+I\left( {\frac{{{V_{b,\tau }}}}{{V_{{b,\tau }}^{{LF}}}}} \right)+P} \right):{\lambda _{P\,b,\tau }}\,\,\forall b,\tau \hfill \\ \end{gathered}$$

3 $$\begin{gathered} {Q_{DS\,b,\tau }}+\sum\limits_{i} {{A_{i,b}}{Q_{CVE\,i,\tau }}} - \sum\limits_{k} {{B_{b,k}}{Q_{DL\,b,k,\tau }}} =\, \hfill \\ \left( {1 - {C_b}} \right){Q_{L\,b,\tau }}+{C_b}{Q_{L\,b,\tau }}\left( {Z{{\left( {\frac{{{V_{b,\tau }}}}{{V_{{b,\tau }}^{{LF}}}}} \right)}^2}+I\left( {\frac{{{V_{b,\tau }}}}{{V_{{b,\tau }}^{{LF}}}}} \right)+P} \right):{\lambda _{Q\,b,\tau }}\,\,\forall b,\tau \hfill \\ \end{gathered}$$

4 $${P_{DL\,b,k,\tau }}={g_{DL\,b,k}}{\left( {{V_{b,\tau }}} \right)^2} - {V_{b,\tau }}{V_{k,\tau }}\left\{ \begin{gathered} {g_{DL\,b,k}}\cos \left( {{\sigma _{b,\tau }} - {\sigma _{k,\tau }}} \right)+ \hfill \\ {b_{DL\,b,k}}\sin \left( {{\sigma _{b,\tau }} - {\sigma _{k,\tau }}} \right) \hfill \\ \end{gathered} \right\}:{\lambda _{PL\,b,k,\tau }}\,\,\,\,\forall b,k,\tau$$

5 $${Q_{DL\,b,k,\tau }}= - {g_{DL\,b,k}}{\left( {{V_{b,\tau }}} \right)^2}+{V_{b,\tau }}{V_{k,\tau }}\left\{ \begin{gathered} {b_{DL\,b,k}}\cos \left( {{\sigma _{b,\tau }} - {\sigma _{k,\tau }}} \right) - \hfill \\ {g_{DL\,b,k}}\sin \left( {{\sigma _{b,\tau }} - {\sigma _{k,\tau }}} \right) \hfill \\ \end{gathered} \right\}:{\lambda _{QL\,b,k,\tau }}\,\,\,\,\forall b,k,\tau$$

6 $${\sigma _{b,\tau }}=0:{\lambda _{\sigma \,b,\tau }}\quad \forall \;b={\text{Slack}}\,{\text{bus}},\tau$$

7 $$\sqrt {{{\left( {{P_{DL\,b,k,\tau }}} \right)}^2}+{{\left( {{Q_{DL\,b,k,\tau }}} \right)}^2}} \leqslant S_{{DL\,b,k}}^{{up}}:{\bar {\mu }_{SL\,b,k,\tau }}\quad \forall b,k,\tau$$

8 $$\sqrt {{{\left( {{P_{DS\,b,\tau }}} \right)}^2}+{{\left( {{Q_{DS\,b,\tau }}} \right)}^2}} \leqslant S_{{DS\,b}}^{{up}}:{\bar {\mu }_{SS\,b,\tau }}\quad \forall b={\text{Slack}}\,{\text{bus}},\tau$$

9 $$V_{b}^{{lo}} \leqslant {V_{b,\tau }} \leqslant V_{b}^{{up}}:{\underline {\mu } _{V\,b,\tau }},{\bar {\mu }_{V\,b,\tau }}\quad \forall b,\tau$$

10 $${P_{VPP\,i,\tau }}={P_{PV\,i,\tau }}+{P_{WT\,i,\tau }}+{P_{DR\,i,\tau }}+\left( {{P_{DCH\,i,\tau }} - {P_{CH\,i,\tau }}} \right) - {P_{L\,i,\tau }}:{\lambda _{VPP\,i,\tau }}\,\,\,\,\,\,\forall i,\tau$$

11 $$- {\psi _i}{P_{L\,i,\tau }} \leqslant {P_{DR\,i,\tau }} \leqslant {\psi _i}{P_{L\,i,\tau }}:{\underline {\mu } _{DR\,i,\tau }},{\bar {\mu }_{DR\,i,\tau }}\,\,\,\,\,\,\forall i,\tau$$

12 $$\sum\limits_{\tau } {{P_{DR\,i,\tau }}=0} :{\lambda _{DR\,i}}\,\,\,\,\,\,\forall i$$

13 $$0 \leqslant {P_{CH\,i,\tau }} \leqslant C{R_i}:{\bar {\mu }_{CH\,i,\tau }}\,\,\,\,\,\,\forall i,\tau$$

14 $$0 \leqslant {P_{DCH\,i,\tau }} \leqslant D{R_i}:{\bar {\mu }_{DCH\,i,\tau }}\,\,\,\,\,\,\forall i,\tau$$

15 $$E_{i}^{{lo}} \leqslant E_{i}^{{in}}+\sum\limits_{{o=1}}^{\tau } {\left( {\eta _{i}^{{CH}}{P_{CH\,i,o}} - \frac{1}{{\eta _{i}^{{DCH}}}}{P_{DCH\,i,o}}} \right)} \leqslant E_{i}^{{up}}:{\underline {\mu } _{ST\,i,\tau }},{\bar {\mu }_{ST\,i,\tau }}\,\,\,\,\,\,\forall i,\tau$$

16 $${P_{CVE\,i,\tau }}={P_{VPP\,i,\tau }}+{P_{LO\,i,\tau }}:{\lambda _{PC\,i,\tau }}\,\,\,\,\,\,\forall i,\tau$$

17 $${P_{LO\,i,\tau }}={\alpha _i}\left| {{P_{CVE\,i,\tau }}} \right|+{\beta _i}\left| {{Q_{CVE\,i,\tau }}} \right|:{\lambda _{LO\,i,\tau }}\,\,\,\,\,\,\forall i,\tau$$

18 $$\sqrt {{{\left( {{P_{CVE\,i,\tau }}} \right)}^2}+{{\left( {{Q_{CVE\,i,\tau }}} \right)}^2}} \leqslant S_{{CVE\,i}}^{{up}}:{\bar {\mu }_{SC\,i,\tau }}\quad \forall i,\tau$$

Equation (1) formulates the objective function of IDN with CVEs. This equation represents the maximization of CVE profits in the energy market (the first part of the equation) and the reactive power market (the second part of the equation)^6^. Profit in each market is equal to the product of the market price and the power of the CVEs associated with that market. If CVEs have a positive value, it means that they are making a profit from the relevant market. However, if they have a negative value, it means that they are purchasing from the relevant market, and Eq. (1) in this case represents the cost. Based on^6^, the reactive power price has been considered a ratio (*K*_*Q*_) of the energy price.

The objective function (1) considers the participation of CVEs in two energy and reactive power markets. The energy market receives the amount of active power offered by CVEs and announces the income or cost of CVEs based on the energy price. The same happens in the reactive power market for CVEs^6^. CVEs announce their reactive power to the reactive power market and earn money from the market based on the price of reactive power. Since the power distribution network generally has inductive loads, CVES must operate in capacitive or reactive power generation mode. CVEs are expected to always earn money from the reactive power market. Accordingly, the first term of Eq. (1), $\sum\nolimits_{{i,\tau }} {{\rho _{E\,\tau }}{P_{CVE\,i,\tau }}}$, refers to the participation of CVEs in the energy market. The second term, $\sum\nolimits_{{i,\tau }} {{\rho _{E\,\tau }}{K_Q}{Q_{CVE\,i,\tau }}}$, refers to the participation of CVEs in the reactive power market^6^.

The AC-OPF limitations in IDN are expressed in (2)-(9)^11,12,14,15^. Constraints (2)-(6) represent the AC power flow (AC-PF) equation IDN buses’ reactive and active power balance^24–27^, power across the distribution line, and the voltage angle of the slack bus^11,12^. In this problem, it is assumed that there are wind and solar renewable resources and CVE in the network. They can inject active power into the grid. In Eq. (2), the active power balance in each network bus includes the active power of the substation and the distribution line, the mentioned elements, and the load. In the load model section, for the buses that have CVE, it is assumed that CVE can adjust the voltage with load control. The load model of constant power, current, and impedance (ZIP) was used for buses that had CVE^18–21^. However, the constant power load model is considered for the buses that do not have CVE. In Eq. (3), which refers to the balance of reactive power in network buses, it is assumed that only CVE can control reactive power^19^. In Eq. (3), there is the reactive power of the distribution line and substation, CVE, and load. In this equation, the ZIP model is considered for buses that have CVE^18^. In Eq. (4)/(5), the amount of active/reactive power passing through a distribution line is calculated. This power depends on the voltage of the buses at the near and far ends of the distribution line^21^. Value for voltage angle in slack bus is presented in Eq. (6). Constraints (7)-(9) represent the operating limits of IDN, i.e., the maximum apparent power limit across the distribution line and distribution substation, as well as the bus voltage limit, respectively^14,15^. Distribution substations are located at the slack bus in these equations to connect the upstream network to IDN. Thus, the variables of *P*_*DS*_ and *Q*_*DS*_ in other buses have a value of zero, and constraint (8) is expressed only for the slack bus. In the context of constraint (9), there is a lower limit that prevents power outages caused by high voltage drop^28–31^ and an upper limit that prevents equipment insulation failure due to overvoltage^15^. It is assumed in the problem that there are two types of critical and non-critical loads. Critical loads are placed on buses whose voltage amplitude variations are not significant. In these buses, the voltage amplitude is generally maintained within the range of [0.98, 1.02]^10^. In non-critical loads, a wider range of voltage amplitude can be tolerated, so that the voltage amplitude range is generally [0.9, 1.05] in these conditions^10^.

The operation model of CVEs is stated in constraints (10)-(18), among which Eqs. (10)-(15) are related to the formulation of VPPs. As provided in (10) of power balance in VPP, VPP includes renewable sources such as photovoltaic and wind system^32–35^. Active power of renewable unit is a parameter^36–39^. Then, the DRP operation model is presented in constraints (11) and (12)^1^. This DRP is an incentive model based on energy prices. In this DRP, a portion of consumers’ energy consumption is shifted from expensive to cheap energy hours. Since expensive (cheap) energy hours generally correspond to the peak (off-peak) period^1^, the DRP requires consumers to provide a percentage of their peak-hour energy consumption during off-peak hours. Thus, constraint (11) indicates the range of changes in active power in DRP. According to constraint (12), any energy savings achieved during peak hours will be achieved during off-peak hours as well^1^. The operation model of storage devices is expressed in (13)-(15)^2^. These equations represent the limits of charge and discharge rates^40–43^, respectively, and the limits of the energy stored in the storage. In stationary storage devices such as batteries, charge and discharge rates (*CR* and *DR*), initial energy (*E*^*in*^), and the minimum and maximum storable energy in storage (*E*^*lo*^ and *E*^*up*^) are independent of time^2^. However, in mobile storage devices such as EVs, these parameters change over time because the number of EVs changes at any moment^44,45^. In the operation model of EVs, these parameters will have an index of *τ*. EVs must store a certain amount of energy during the day. Based on what we have already observed in this paper, the batteries of EVs are likely to be fully charged^44^. Accordingly, constraint (15) for EVs is modeled as $E_{{i,\tau }}^{{lo}} \leqslant E_{{i,\tau }}^{{in}}+\sum\nolimits_{{o=1}}^{\tau } {( {\eta _{i}^{{CH}}{P_{CH\,i,o}} - \frac{1}{{\eta _{i}^{{DCH}}}}{P_{DCH\,i,o}}} )} =E_{{i,\tau }}^{{up}}$. Note that at moment *τ*, parameter values *CR*, *DR*, and Elo for EVs equal charge rate plus voltage, discharge rate, and minimum energy of EVs connected to the network at this time, respectively. The value of *E*^*in*^ at moment *τ* is equal to the total energy at the start of the newly joined EVs at moment *τ*. The value of *E*^*up*^ at moment *τ* is equal to the sum of energy consumption required by EVs disconnected from the network at that moment. Considering the full charge for each EV, its power consumption will be equal to its battery capacity^45^. The following is the formulation of ES in constraints (16)-(18). Note that *P*_*CEV*_ is on the IDN side and *P*_*VPP*_ is on the VPP output side. There are ES losses between these two powers. So, the active power of CVE as (16) will be equal to the sum of the active power of VPP and the active losses of ES. In constraint (17), the ES loss is calculated, which is a factor of the absolute value of the active and reactive power of CVE^10^. Finally, the ES capacity limit is presented in constraint (18), a measure of how much power appears to be flowing through ES at any given time. Note that, as in Fig. 1, there is a controllable load at the ES output, which is used for voltage regulation. In this paper, as in^10^, it is modeled as an impedance, current, and constant power (ZIP) load, which appears on the left side of Eqs. (2) and (3)^21^. Finally, expressions *λ* and *µ* represent the dual variables of different constraints.

The electric inverter has a DC/AC converter, which is equipped with an insulated-gate bipolar transistor (IGBT) bridge. IGBTs with proper switching can control the active and reactive power passing through them. Equations (16)-(18) have been used in the proposed problem. Since it can control the active and reactive power of the network, it works to obtain financial benefits for providing such services based on the plan of the electricity market. Their participation in the energy and reactive power market is considered. Problem Eqs. (1)-(18) are based on this assumption.

According to problem Eqs. (1)-(18), the optimal performance of the ES coupler and VPP system should be such that the power distribution network constraints are met. In the objective function of this problem, the request of the VPP operator from the energy and reactive power market in exchange for its own energy and reactive power management in the power distribution network is considered. Based on Fig. 1, it can be seen that the operator of this system is in communication with the network operator, then it determines its optimal performance based on the network constraints. For example, the network operator confirms whether the injected/received power of the system from the power distribution network leads to the destruction of the network restrictions or not. If the power of the system does not lead to damage to network restrictions, it is approved. This power is provided to the market operator. The market operator calculates the profit of the system based on the price signal.

It is noteworthy that the main factor in reducing the flexibility of a system is the presence of renewable resources in that system because they do not have control over their active power output, and since the output power of these resources is dependent on renewable phenomena, the output power is uncertain, which leads to the reduction of flexibility in the system. Nonetheless, flexible sources such as storage devices, demand response, and electric vehicles can compensate for the power fluctuations of renewable sources as they can control their active power. Since a VPP has flexible resources in the proposed scheme, these resources can both improve the flexibility of the VPP and enable the VPP to control the active power. VPP in the power distribution network can compensate for the fluctuations of renewable resources to improve the flexibility of the distribution network. It is noteworthy that part of the capacity of flexibility resources is used to promote flexibility, but the other part of its capacity is used for other purposes, such as the economic and technical purposes of the network and VPP.

### Linear approximation model

Equations (1)−(18) are in the non-convex nonlinear programming (NLP) form (due to AC-PF constraints^46,47^). The solvers of this problem generally do not have the same solutions^6^, so the coefficient of confidence in response to this problem is low. This formulation is an operation problem. In operation problems, the execution step is generally small, so the low computational time is of particular importance^6^. However, NLP solvers generally rely on iterative numerical methods that take a long time to compute^48–52^. Based on the linear approximation model presented in this section, it is possible to produce a unique solution in a short time^2^.

To linearize AC-OPF constraints, it should be noted that according to^6^, the voltage angle deviation at the two sending and receiving end buses of a distribution line for a distribution network is generally less than 6 degrees. The expressions $\cos \left( {{\sigma _{b,\tau }} - {\sigma _{k,\tau }}} \right)$ and $\sin \left( {{\sigma _{b,\tau }} - {\sigma _{k,\tau }}} \right)$ are approximated to 1 and $\left( {{\sigma _{b,\tau }} - {\sigma _{k,\tau }}} \right)$, respectively. The conventional piecewise linearization technique is used to linearize the expression of the squared voltage amplitude and the product of the voltage amplitudes. In this method, the variable *V* is expressed as $\sum\limits_{j} {\Delta {V_j}}$, where *ΔV* and *j* represent the voltage deviation and the piecewise index, respectively. In this case, based on^1^, *V*^*2*^ is formulated as ${\left( {V_{{}}^{{lo}}} \right)^2}+\sum\limits_{j} {s{l_j}\Delta {V_j}}$, and *V*_*b*_*V*_*k*_ will be equal to ${\left( {V_{{}}^{{lo}}} \right)^2}+V_{{}}^{{lo}}\sum\limits_{j} {\left( {\Delta {V_{b,j}}+\Delta {V_{k,j}}} \right)}$. So, the variable *ΔV* replaces the variable *V* in AC-OPF, whose maximum value is equal to the ratio of the difference between *V*^*lo*^ and *V*^*up*^ to the number of linear pieces (*n*_*J*_). The linearized formulation of constraints (2)-(5) and (9) can be written as (19)-(23), respectively. With AC-OPF linearization, it is known as linearized AC-OPF (LAC-OPF).

19 $$\begin{gathered} {P_{DS\,b,\tau }}+{P_{PV\,b,\tau }}+{P_{WT\,b,\tau }}+\sum\limits_{i} {{A_{i,b}}{P_{CVE\,i,\tau }}} - \sum\limits_{k} {{B_{b,k}}{P_{DL\,b,k,\tau }}} =\, \hfill \\ \,\,\,\,\,\,\,\,\,\,\,\left( {1 - {C_b}} \right){P_{L\,b,\tau }}+{C_b}{P_{L\,b,\tau }}\left( {Z\left( {\frac{{{{\left( {V_{b}^{{lo}}} \right)}^2}+\sum\limits_{j} {s{l_j}\Delta {V_{b,\tau ,j}}} }}{{{{\left( {V_{{b,\tau }}^{{LF}}} \right)}^2}}}} \right)+I\left( {\frac{{\sum\limits_{j} {\Delta {V_{b,\tau ,j}}} }}{{V_{{b,\tau }}^{{LF}}}}} \right)+P} \right):{{\lambda ^{\prime}}_{P\,b,\tau }}\,\,\forall b,\tau \hfill \\ \end{gathered}$$

20 $$\begin{gathered} {Q_{DS\,b,\tau }}+\sum\limits_{i} {{A_{i,b}}{Q_{CVE\,i,\tau }}} - \sum\limits_{k} {{B_{b,k}}{Q_{DL\,b,k,\tau }}} = \hfill \\ \,\,\,\,\left( {1 - {C_b}} \right){Q_{L\,b,\tau }}+{C_b}{Q_{L\,b,\tau }}\left( {Z\left( {\frac{{{{\left( {V_{b}^{{lo}}} \right)}^2}+\sum\limits_{j} {s{l_j}\Delta {V_{b,\tau ,j}}} }}{{{{\left( {V_{{b,\tau }}^{{LF}}} \right)}^2}}}} \right)+I\left( {\frac{{\sum\limits_{j} {\Delta {V_{b,\tau ,j}}} }}{{V_{{b,\tau }}^{{LF}}}}} \right)+P} \right):{{\lambda ^{\prime}}_{Q\,b,\tau }}\,\,\forall b,\tau \hfill \\ \end{gathered}$$

21 $$\begin{gathered} {P_{DL\,b,k,\tau }}={g_{DL\,b,k}}\sum\limits_{j} {\left( {\left( {s{l_j} - V_{b}^{{lo}}} \right)\Delta {V_{b,\tau ,j}} - V_{b}^{{lo}}\Delta {V_{k,\tau ,j}}} \right)} - {\left( {V_{b}^{{lo}}} \right)^2}{b_{DL\,b,k}}\left( {{\sigma _{b,\tau }} - {\sigma _{k,\tau }}} \right):{{\lambda ^{\prime}}_{PL\,b,k,\tau }} \hfill \\ \quad \forall b,k,\tau \hfill \\ \end{gathered}$$

22 $$\begin{gathered} {Q_{DL\,b,k,\tau }}= - {g_{DL\,b,k}}\sum\limits_{j} {\left( {\left( {s{l_j} - V_{b}^{{lo}}} \right)\Delta {V_{b,\tau ,j}} - V_{b}^{{lo}}\Delta {V_{k,\tau ,j}}} \right)} - {\left( {V_{b}^{{lo}}} \right)^2}{g_{DL\,b,k}}\left( {{\sigma _{b,\tau }} - {\sigma _{k,\tau }}} \right):{{\lambda ^{\prime}}_{QL\,b,k,\tau }} \hfill \\ \quad \forall b,k,\tau \hfill \\ \end{gathered}$$

23 $$0 \leqslant \Delta {V_{b,\tau ,j}} \leqslant \frac{{V_{b}^{{up}} - V_{b}^{{lo}}}}{{{n_J}}}:{\bar {\mu }_{\Delta V\,b,\tau ,j}}\quad \forall b,\tau ,j$$

Based on constraints (7)–(8) and (18), the PQ coordinates of a circular plane have a center of origin and a radius S, i.e., $\sqrt {{P^2}+{Q^2}} \leqslant S$. To linearize this equation, a circular plane is approximated to a plane in the form of a regular polygon with *n*_*P*_ sides^2^. Each side of this plane has a linear relationship in the form of $P \times \cos \left( {\omega \times \Delta \delta } \right)+Q \times \sin \left( {\omega \times \Delta \delta } \right)=S$^2^ in which *ω* represents the side that varies between 1 and *n*_*P*_. In addition, *Δδ* indicates an angle deviation of 360/*n*_*P*_. Next, from each side, a square plate can be extracted as $P \times \cos \left( {\omega \times \Delta \delta } \right)+Q \times \sin \left( {\omega \times \Delta \delta } \right) \leqslant S$; considering the planes from all sides in the proposed problem, a plate is extracted in the form of a regular polygon^2^. Constraints (7)-(8) and (18) are linearized as (24)-(26), respectively.

24 $${P_{DL\,b,k,\tau }}\cos \left( {\omega \times \Delta \delta } \right)+{Q_{DL\,b,k,\tau }}\sin \left( {\omega \times \Delta \delta } \right) \leqslant S_{{DL\,b,k}}^{{up}}:{\bar {\mu }^{\prime}_{SL\,b,k,\tau ,\omega }}\quad \forall b,k,\tau ,\omega$$

25 $${P_{DS\,b,\tau }}\cos \left( {\omega \times \Delta \delta } \right)+{Q_{DS\,b,\tau }}\sin \left( {\omega \times \Delta \delta } \right) \leqslant S_{{DS\,b}}^{{up}}:{\bar {\mu }^{\prime}_{SS\,b,\tau ,\omega }}\quad \forall b={\text{Slack}}\,{\text{bus}},\tau ,\omega$$

26 $${P_{CVE\,i,\tau }}\cos \left( {\omega \times \Delta \delta } \right)+{Q_{CVE\,i,\tau }}\sin \left( {\omega \times \Delta \delta } \right) \leqslant S_{{CVE\,i}}^{{up}}:{\bar {\mu }^{\prime}_{SC\,i,\tau ,\omega }}\quad \forall i,\tau ,\omega$$

In constraint (17), the nonlinear expression is the resultant of the active and reactive power of CVE. Since the power distribution network generally has an ohmic-inductive load, CVE is expected to operate to control reactive power in the capacitive mode (reactive power generation). The phrase |*Q*_*CEV*_| will be *Q*_*CEV*_. Yet, to linearize |*P*_*CVE*_|, the variable *P*_*CVE*_ is first divided into positive ($P_{{CVE}}^{+}$) and negative ($P_{{CVE}}^{ - }$) components, which have positive values and contain the positive and negative values of the *P*_*CVE*_ variable, respectively. As a result, *P*_*CVE*_ is equal to the difference of$P_{{CVE}}^{+}$and$P_{{CVE}}^{ - }$, and |*P*_*CVE*_| is equal to the sum of the positive and negative components of the active power of CVE. Accordingly, constraint (17) is linearized as constraints (27)-(30), where (27) represents the linearized model of constraint (17). In (28), *P*_*CVE*_ is expressed in terms of positive and negative components. The limitations of these components are stated in (29) and (30).

27 $${P_{LO\,i,\tau }}={\alpha _i}\left( {P_{{CVE\,i,\tau }}^{+}+P_{{CVE\,i,\tau }}^{ - }} \right)+{\beta _i}{Q_{CVE\,i,\tau }}:{\lambda ^{\prime}_{LO\,i,\tau }}\,\,\,\,\,\,\forall i,\tau$$

28 $${P_{CVE\,i,\tau }}=P_{{CVE\,i,\tau }}^{+} - P_{{CVE\,i,\tau }}^{ - }:{\lambda _{C\,i,\tau }}\,\,\,\,\,\,\forall i,\tau$$

29 $$0 \leqslant P_{{CVE\,i,\tau }}^{+} \leqslant S_{{CVE\,i}}^{{up}}:{\bar {\mu }_{+\,i,\tau }}\,\,\,\,\,\,\forall i,\tau$$

30 $$0 \leqslant P_{{CVE\,i,\tau }}^{ - } \leqslant S_{{CVE\,i}}^{{up}}:{\bar {\mu }_{ - \,i,\tau }}\,\,\,\,\,\,\forall i,\tau$$

Finally, the linearized model of the suggested scheme can be stated as (31)-(32):

31 $$\hbox{max} \,\,\,\,\,\,F=\sum\limits_{{i,\tau }} {{\rho _{E\,\tau }}\left( {{P_{CVE\,i,\tau }}+{K_Q}{Q_{CVE\,i,\tau }}} \right)}$$

Subject to

32 $${\text{Constraints }}\left( {\text{6}} \right),{\text{ }}\left( {{\text{1}}0} \right) - \left( {{\text{16}}} \right),{\text{ and }}\left( {{\text{19}}} \right) - \left( {{\text{3}}0} \right)$$

## Robust flexible operation of CEVs in IDN

In problems (31)-(32), there is uncertainty in the parameters of load, renewable power, energy price, energy, and the charge/discharge rates of EVs. According to the proposed plan, there are many uncertain parameters, so a significant number of scenarios is required to model them using stochastic or probabilistic programming. This increases the computational time. However, low computational time is necessary for operation problems^6^. Additionally, in stochastic or probabilistic modeling, different uncertainties require different probability distribution functions (PDFs). Accessing an accurate PDF requires long-term statistical studies (for example, one year), which is very time-consuming^53^. To address these challenges and bridge the third research gap, robust optimization is adopted to model the uncertainties in this section. This method has only one scenario, equivalent to the worst-case scenario resulting from uncertainties^54^. Since it involves only one scenario, the computational time for problem-solving is lower than that of stochastic and probabilistic modeling. The optimal solution^55–59^ obtained in the worst-case scenario is robust against predictive uncertainties^60^. Thus, this method can enhance the robustness of CVEs in improving IDN operation amid the prediction errors of the mentioned uncertainties.

It is noteworthy that load, energy price, renewable power, and electric vehicles exhibit uncertainty. In other words, these parameters have a definite value at a certain moment. For this purpose, different scenarios for these uncertainties must be included in the proposed problem, with each scenario representing a different level of uncertainty. This increases both the problem size and computational time. However, low computational time is necessary for operation problems. In this article, adaptive robust optimization was adopted to model uncertainties. This method considers only one scenario, reducing both the problem size and computational time. In this method, the mentioned scenario has the values of the mentioned uncertainties to achieve the worst situation for the objective function. This scenario is referred to as the worst-case scenario. In the robust method, an optimal point is obtained under the worst-case scenario, making it the most resistant solution to uncertainty prediction errors. If another scenario occurs, it must represent an improved situation compared to the worst-case scenario. So, in this work, ARO was used to model uncertainties, serving to find a robust solution and identify the worst-case scenario for the mentioned uncertainties.

### Uncertainty in the proposed scheme

According to the proposed plan, parameters of load (*P*_*L*_ and *Q*_*L*_), renewable power (*P*_*PV*_ and *Q*_*PV*_), energy price (*ρ*_*E*_), the charge and discharge rates of EVs (*CR* and *DR*), the initial energy of EVs (*E*^*in*^), and minimum and maximum stored energy in EVs (*E*^*ol*^ and *E*^*up*^) are uncertain. In this section, these parameters are modeled by ARO, which is an accurate method that simultaneously obtains the robust optimal solution and the worst-case scenario. In this method, it is, first, necessary to estimate the set of uncertainties, which is required to extract the matrix of uncertainties. As a result of the proposed scheme, we obtain the matrix of uncertain parameters ($\bar {u}$) (33), which has the predicted values ​​of uncertainties. In this matrix, the row count is *n*_*τ*_ (total number of operating hours), and the column count (*n*_*c*_) is 4×(*n*_*b*_ + *n*_*i*_) + 5×*n*_*i*_ + 1 where *n*_*b*_ and *n*_*i*_, represent the number of buses and CVEs located in the network, respectively. Uncertain matrix (*u*) can be written as (34) in which these uncertainties are considered variables.

33 $$\bar {u}={\left[ {{P_L}\,\,{Q_L}\,\,{P_{PV}}\,\,{P_{WT}}\,\,CR\,\,DR\,\,{E^{lo}}\,\,{E^{in}}\,\,{E^{up}}\,\,{\rho _E}} \right]^T}$$

34 $$u={\left[ {P_{L}^{u}\,\,Q_{L}^{u}\,\,P_{{PV}}^{u}\,\,P_{{WT}}^{u}\,\,C{R^u}\,\,D{R^u}\,\,{E^{lo,u}}\,\,{E^{in,u}}\,\,{E^{up,u}}\,\,\rho _{E}^{u}} \right]^T}$$

The variations in the range of the uncertain variables are based on a set of uncertainties. This set for row *τ* of a matrix of uncertain variables can be written as follows^53^:

35 $${U_\tau }=\left\{ {{u_\tau } \in {R^{{n_c}}}|\frac{1}{{{n_c}}}\sum\limits_{{h=1}}^{{{n_c}}} {\frac{{\left| {{u_{\tau ,h}} - {{\tilde {u}}_{\tau ,h}}} \right|}}{{{{\bar {u}}_{\tau ,h}}}}} \leqslant B{U_\tau },\,\,\forall {u_\tau } \in \left[ {{{\bar {u}}_{\tau ,h}} - {{\tilde {u}}_{\tau ,h}},{{\bar {u}}_{\tau ,h}}+{{\tilde {u}}_{\tau ,h}}} \right]} \right\}\,\,\,\,\,\forall \tau$$

where $\tilde {u}$ represents uncertainty deviation and *BU* represents the budget of uncertainty, which varies between 0 and 1 in which 0 means that each uncertain variable has the same value as its predicted value; that is, it is deterministic modeling. However, if its value is greater than 0, it means that some parameters have uncertainty, so its value of 1 means that all these parameters are uncertain^53^. Finally, the term $\left[ {\bar {u} - \tilde {u},\bar {u}+\tilde {u}} \right]$indicates the range of changes in the uncertain variable.

### ARO model of the proposed scheme

This section describes how the worst-case situation and robust solution are achieved by ARO at the same time. According to^53^, the robust model for max_y∈Φ(y)_ *f(y)* is min_*u*_ max_y∈Φ(y, u)_ *f(y)*, where *y* and *u* denote the original and uncertain variables. *Φ(y*,* u)* is defined as {*g*_1_(*y*) *=* 0, *g*_2_(*y*) *= u*,* s*_1_(*y*) *≤ 0*,* s*_2_(*y*) *≤ u*}. In *Φ*(*y*) for max_y∈Φ(y)_ *f(y)*, *u* denotes constant values shown by$\bar {u}$. In min_*u*_ max_y∈Φ(y, u)_ *f(y)*,* u* is a variable. The dual of max_y∈Φ(y, u)_ *f(y)* needs to be found in the first step of solving the robust model. When the robust model is achieved, it is transformed into a min-min form. Thereby, the robust model of the linear deterministic expression for Eqs. (31) and (32) can be stated as:

36 $$\begin{gathered} \hbox{min} \,\,\,\,\,G=\sum\limits_{{b,\tau }} {\left\{ \begin{gathered} \left( {\left( {1 - {C_b}} \right)P_{{L\,b,\tau }}^{u}+{C_b}P_{{L\,b,\tau }}^{u}\left( {Z\left( {\frac{{V_{b}^{{lo}}}}{{V_{{b,\tau }}^{{LF}}}}} \right)+P} \right) - P_{{PV\,b,\tau }}^{u} - P_{{WT\,b,\tau }}^{u}} \right){{\lambda ^{\prime}}_{P\,b,\tau }}+ \hfill \\ \left( {\left( {1 - {C_b}} \right)Q_{{L\,b,\tau }}^{u}+{C_b}Q_{{L\,b,\tau }}^{u}\left( {Z\left( {\frac{{V_{b}^{{lo}}}}{{V_{{b,\tau }}^{{LF}}}}} \right)+P} \right)} \right){{\lambda ^{\prime}}_{Q\,b,\tau }}+\sum\limits_{j} {\frac{{V_{b}^{{up}} - V_{b}^{{lo}}}}{{{n_J}}}{{\bar {\mu }}_{\Delta V\,b,\tau ,j}}} \hfill \\ \end{gathered} \right\}} \hfill \\ \,\,\,\,\,\,\,\,\,\,\,\,\,\,\,\,\,\,\,\,+\sum\limits_{{\tau ,\omega }} {\left\{ {S_{{DS\,b={\text{Slack}}\,{\text{bus}}}}^{{up}}{{\bar {\mu }^{\prime}}_{SS\,b={\text{Slack}}\,{\text{bus}},\tau ,\omega }}+\sum\limits_{{b,k}} {S_{{DL\,b,k}}^{{up}}{{\bar {\mu }^{\prime}}_{SL\,b,k,\tau ,\omega }}} +\sum\limits_{i} {S_{{CVE\,i}}^{{up}}{{\bar {\mu }^{\prime}}_{SC\,i,\tau ,\omega }}} } \right\}} + \hfill \\ \,\,\,\,\,\,\,\,\,\,\,\,\,\,\,\,\,\,\,\,\,\,\,\,\sum\limits_{{i,\tau }} {\left\{ \begin{gathered} S_{{CVE\,i}}^{{up}}{{\bar {\mu }}_{+\,i,\tau }}+S_{{CVE\,i}}^{{up}}{{\bar {\mu }}_{ - \,i,\tau }}+\left( {P_{{PV\,i,\tau }}^{u}+P_{{WT\,i,\tau }}^{u} - P_{{L\,i,\tau }}^{u}} \right){\lambda _{VPP\,i,\tau }} - {\psi _i}P_{{L\,i,\tau }}^{u}{\underline {\mu } _{DR\,i,\tau }}+ \hfill \\ {\psi _i}P_{{L\,i,\tau }}^{u}{{\bar {\mu }}_{DR\,i,\tau }}+CR_{{i,\tau }}^{u}{{\bar {\mu }}_{CH\,i,\tau }}+DR_{{i,\tau }}^{u}{{\bar {\mu }}_{DCH\,i,\tau }}+\left( {E_{{i,\tau }}^{{lo,u}} - E_{{i,\tau }}^{{in,u}}} \right){\underline {\mu } _{ST\,i,\tau }}+ \hfill \\ \left( {E_{{i,\tau }}^{{up,u}} - E_{{i,\tau }}^{{in,u}}} \right){{\bar {\mu }}_{ST\,i,\tau }} \hfill \\ \end{gathered} \right\}} \hfill \\ \end{gathered}$$

Subject to:

37 $${\lambda ^{\prime}_{P\,b,\tau }}+\sum\limits_{\omega } {\cos \left( {\omega \times \Delta \delta } \right){{\bar {\mu }^{\prime}}_{SS\,b,\tau ,\omega }}} =0:{P_{DS\,b,\tau }}\,\,\,\,\,\,\forall b={\text{Slack}}\,{\text{bus}},\tau$$

38 $${\lambda ^{\prime}_{Q\,b,\tau }}+\sum\limits_{\omega } {\sin \left( {\omega \times \Delta \delta } \right){{\bar {\mu }^{\prime}}_{SS\,b,\tau ,\omega }}} =0:{Q_{DS\,b,\tau }}\,\,\,\,\,\,\forall b={\text{Slack}}\,{\text{bus}},\tau$$

39 $$- {B_{b,k}}{\lambda ^{\prime}_{P\,b,\tau }}+{\lambda ^{\prime}_{PL\,b,k,\tau }}+\sum\limits_{\omega } {\cos \left( {\omega \times \Delta \delta } \right){{\bar {\mu }^{\prime}}_{SL\,b,k,\tau ,\omega }}} =0:{P_{DL\,b,k,\tau }}\,\,\,\,\,\,\forall b,k,\tau$$

40 $$- {B_{b,k}}{\lambda ^{\prime}_{Q\,b,\tau }}+{\lambda ^{\prime}_{QL\,b,k,\tau }}+\sum\limits_{\omega } {\sin \left( {\omega \times \Delta \delta } \right){{\bar {\mu }^{\prime}}_{SL\,b,k,\tau ,\omega }}} =0:{Q_{DL\,b,k,\tau }}\,\,\,\,\,\,\forall b,k,\tau$$

41 $${\lambda _{PC\,i,\tau }}+{\lambda _{C\,i,\tau }}+\sum\limits_{b} {{A_{i,b}}{{\lambda ^{\prime}}_{P\,b,\tau }}} +\sum\limits_{\omega } {\cos \left( {\omega \times \Delta \delta } \right){{\bar {\mu }^{\prime}}_{SC\,i,\tau ,\omega }}} =\rho _{{E\,\tau }}^{u}:{P_{CVE\,i,\tau }}\,\,\,\,\,\,\forall i,\tau$$

42 $$- {\alpha _i}{\lambda ^{\prime}_{LO\,i,\tau }} - {\lambda _{C\,i,\tau }}+{\bar {\mu }_{+\,i,\tau }} \leqslant 0:P_{{CVE\,i,\tau }}^{+}\,\,\,\,\,\,\forall i,\tau$$

43 $$- {\alpha _i}{\lambda ^{\prime}_{LO\,i,\tau }}+{\lambda _{C\,i,\tau }}+{\bar {\mu }_{ - \,i,\tau }} \leqslant 0:P_{{CVE\,i,\tau }}^{ - }\,\,\,\,\,\,\forall i,\tau$$

44 $${\lambda ^{\prime}_{LO\,i,\tau }} - {\lambda _{PC\,i,\tau }} \leqslant 0:{P_{LO\,i,\tau }}\,\,\,\,\,\,\forall i,\tau$$

45 $${\beta _i}{\lambda ^{\prime}_{LO\,i,\tau }}+\sum\limits_{b} {{A_{i,b}}{{\lambda ^{\prime}}_{Q\,b,\tau }}} +\sum\limits_{\omega } {\sin \left( {\omega \times \Delta \delta } \right){{\bar {\mu }^{\prime}}_{SC\,i,\tau ,\omega }}} \geqslant {K_Q}\rho _{{E\,\tau }}^{u}:{Q_{CVE\,i,\tau }}\,\,\,\,\,\,\forall i,\tau$$

46 $${\lambda _{VPP\,i,\tau }} - {\lambda _{PC\,i,\tau }}=0:{P_{VPP\,i,\tau }}\,\,\,\,\,\,\forall i,\tau$$

47 $$- {\lambda _{VPP\,i,\tau }} - {\underline {\mu } _{DR\,i,\tau }}+{\bar {\mu }_{DR\,i,\tau }}+{\lambda _{DR\,i}}=0:{P_{DR\,i,\tau }}\,\,\,\,\,\,\forall i,\tau$$

48 $${\lambda _{VPP\,i,\tau }}+{\bar {\mu }_{CH\,i,\tau }}+\eta _{i}^{{CH}}\left( {{{\bar {\mu }}_{ST\,i,\tau }} - {{\underline {\mu } }_{ST\,i,\tau }}} \right) \leqslant 0:{P_{CH\,i,\tau }}\,\,\,\,\,\,\forall i,\tau$$

49 $$- {\lambda _{VPP\,i,\tau }}+{\bar {\mu }_{DCH\,i,\tau }} - \frac{1}{{\eta _{i}^{{DCH}}}}\left( {{{\bar {\mu }}_{ST\,i,\tau }} - {{\underline {\mu } }_{ST\,i,\tau }}} \right) \leqslant 0:{P_{DCH\,i,\tau }}\,\,\,\,\,\,\forall i,\tau$$

50 $$\begin{gathered} - \left( {Z \times s{l_j}+I} \right){{\lambda ^{\prime}}_{P\,b,\tau }} - \left( {Z \times s{l_j}+I} \right){{\lambda ^{\prime}}_{Q\,b,\tau }}+{{\bar {\mu }}_{\Delta V\,b,\tau ,j}} - {g_{DL\,b,k}}\sum\limits_{j} {\left( {\left( {s{l_j} - V_{b}^{{lo}}} \right){{\lambda ^{\prime}}_{PL\,b,k,\tau }} - V_{b}^{{lo}}{{\lambda ^{\prime}}_{PL\,k,b,\tau }}} \right)} \hfill \\ \,\,\,\,\,\,\,\,\,\,\,\,\,\,\,\,\,\,\,\,\,\,\,\,\,\,\,\,\,\,\,\,\,\,\,\,\,\,\,\,\,\,\,\,\,\,\,\,\,\,\,\,\,\,+{b_{DL\,b,k}}\sum\limits_{j} {\left( {\left( {s{l_j} - V_{b}^{{lo}}} \right){{\lambda ^{\prime}}_{QL\,b,k,\tau }} - V_{b}^{{lo}}{{\lambda ^{\prime}}_{qL\,k,b,\tau }}} \right)} \leqslant 0:\Delta {V_{b,\tau ,j}}\,\,\,\,\,\,\forall b,\tau ,j \hfill \\ \end{gathered}$$

51 $${\left( {V_{b}^{{lo}}} \right)^2}\left( {{b_{DL\,b,k}}\left( {{{\lambda ^{\prime}}_{PL\,b,k,\tau }} - {{\lambda ^{\prime}}_{PL\,k,b,\tau }}} \right)+{g_{DL\,b,k}}\left( {{{\lambda ^{\prime}}_{QL\,b,k,\tau }} - {{\lambda ^{\prime}}_{QL\,k,b,\tau }}} \right)} \right)+{z_b}{\lambda _{\sigma \,b,\tau }}=0:{\sigma _{b,\tau }}\,\,\,\,\,\,\forall b,\tau$$

52 $$\lambda \in \left( { - \infty ,+\infty } \right),\,\,\,\underline {\mu } \leqslant 0,\,\,\,\,\bar {\mu } \geqslant 0$$

53 $$u \in \bigcup\limits_{\tau } {{U_\tau }}$$

The dual form of the linear deterministic model of (31)-(32) is given in (36)-(53). The objective function of the dual or robust problem is shown in (36). Dual constraints of special variables in the main problem are stated in (37)-(51)^53^. Equations (37)-(38) are related to the slack bus because variables *P*_*DS*_ and *Q*_*DS*_ for the rest of the buses are equal to zero. Equation (52) shows the range of the dual variable and is constrained by the main problem. Equation (53) is appended to the robust problem to turn it into a dual problem, showing the uncertainty limit.

It is worth noting that problems (36)-(53) are nonlinear, and their objective functions^61–64^ are quadratic (that is, they have the product of two continuous variables). Their constraints^65–67^ are, also, linear. This is a convex problem, and linear model solvers can solve solving it^68^. A linear equation is not derived for the objective function (36). It should be noted that to implement the proposed optimization problem on a network, the network must be equipped with an intelligent platform such as smart algorithms and telecommunication devices^69–73^.

### The proposed scheme based on the flexibility market model

It is worth noting that DA and RT operations in IDN with renewable sources are not identical due to the uncertainty in power generation from these sources. RT operations may be affected by demand-supply imbalances, which indicate the low flexibility of IDN^3,10^. To improve IDN flexibility, it is necessary to have flexibility sources within IDN, which can quickly control their active power^10^. This holds for CVE because the time constant of ES operation is low due to its electrical circuits, allowing it to control the active power passing through it. Flexibility sources can enhance flexibility by compensating for fluctuations in renewable power during RT operations compared to DA operations. To model the flexibility of a flexible source (CVE), in comparison to the deterministic model scenario, its active power needs to be measured (with the predicted value of uncertainties)^10^. If these changes have a positive (negative) value, CVE operates in an upward (downward) mode. These changes are referred to as flexibility, which has a positive value in both modes of operation^10^. Since CVE enhances IDN flexibility in both modes, it can benefit from the flexibility market. Each mode of CVE provides flexibility equal to the product of power and flexibility price^74^. The modeling of the proposed scheme, considering the flexibility market, can be written as follows:

54 $$\hbox{max} \,\,\,\,\,{F_1}=\sum\limits_{{i,\tau }} {\left( {{\varphi _{U\,\tau }}{F_{U\,i,\tau }}+{\varphi _{L\,\tau }}{F_{L\,i,\tau }}} \right)} - G$$

Subject to:

55 $${F_{U\,i,\tau }} - {F_{L\,i,\tau }}={P_{CVE\,i,\tau }}\left( {\tilde {u} \ne 0} \right) - {P_{CVE\,i,\tau }}\left( {\tilde {u}=0} \right)\,\,\,\,\,\,\forall i,\tau ,{P_{CVE\,i,\tau }}\left( {\tilde {u}=0} \right) \in \arg \left\{ {Eqs.(31) - (32)} \right\}$$

56 $${F_{U\,i,\tau }},{F_{L\,i,\tau }} \geqslant 0\,\,\,\,\,\,\forall i,\tau$$

57 $${\text{Constraints }}\left( {{\text{37}}} \right) - \left( {{\text{53}}} \right)$$

Equation (54) indicates the maximization of CVE profit in the flexibility market (the first part of the equation) and in the energy and reactive power markets (− *G*). It must be noted that min *G* is equal to max *F*, so the expression − *G* appears in (54). Constraint (55) is used to calculate the flexibility of CVEs in upward and downward modes. Like constraint (56), the value of this power is always positive in these two modes of operation. Another constraint of this problem is the same constraints (37)-(53) based on (57).

### Solution process based on the BD algorithm

The robust problem, described by (54)-(57), is solved in this section using the BD method^75^. The main purpose of using the BD method is, in general, to speed up solving the mixed-integer linear problem. It can assist in solving single-stage linear and nonlinear problems subject to complex constraints^75^. Such constraints of the robust model given in this paper are Eqs. (50) and (51). The BD method divides the difficulty into two parts: the master problem (MP) and the sub-problem (SP). In the former, Eq. (54) expresses the objective function, while (37)-(49), (52), (53), (55), and (56) denote the constraints of the master problem. On the other hand, in the latter, Eqs. (50) and (51) are sub-problems (SP), and this model is examined with the help of solutions found by the BD in the master problem:

58 $$\hbox{max} \;\quad er= - \sum \zeta$$

Subject to:

59 $$Left\,side\,\,of\,Eq.(50)+{\zeta _{b,\tau ,j,1}} - {\zeta _{b,\tau ,j,2}}=0:{\pi _{b,\tau ,j,1}}\quad \forall \;b,\tau ,j$$

60 $$Left\,side\,\,of\,Eq.(51)+{\zeta _{b,\tau ,3}} - {\zeta _{b,\tau ,4}}=0:{\pi _{b,\tau ,2}}\quad \forall \;b,\tau$$

61 $$\zeta \geqslant 0$$

in which *ζ* and *π* show the slack and dual variables, respectively. As per the BD method^75^, if *|er|≤ε* (where *ε* is the convergence tolerance level of BD), problems (54), (37)–(49), (52)-(53), and (55)-(56) are solved, while if *|er|≥ε*, in order to make the Benders cut, we must add it to the master problem as follows^75^:

62 $$\sum {{\pi _1} \times Left\,side\,\,of\,Eq.(50)} +\sum {{\pi _2} \times Left\,side\,\,of\,Eq.(51)}$$

**Fig. 2:**
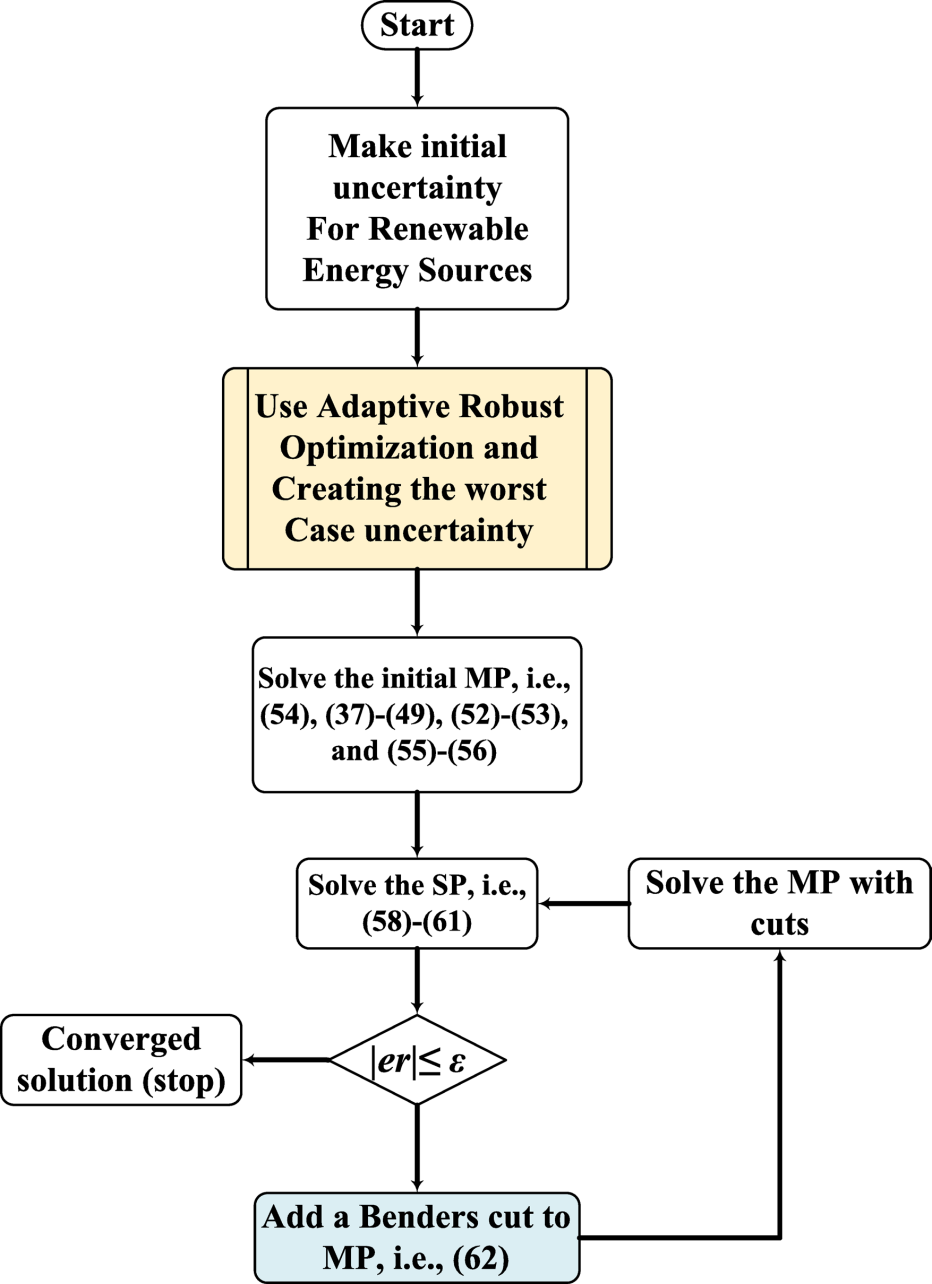
The flowchart of the solution procedure for the proposed problem based on the BD algorithm.

Figure 2 displays the flowchart illustrating how the robust problem is solved.

## Numerical results

### Case study

**Fig. 3:**
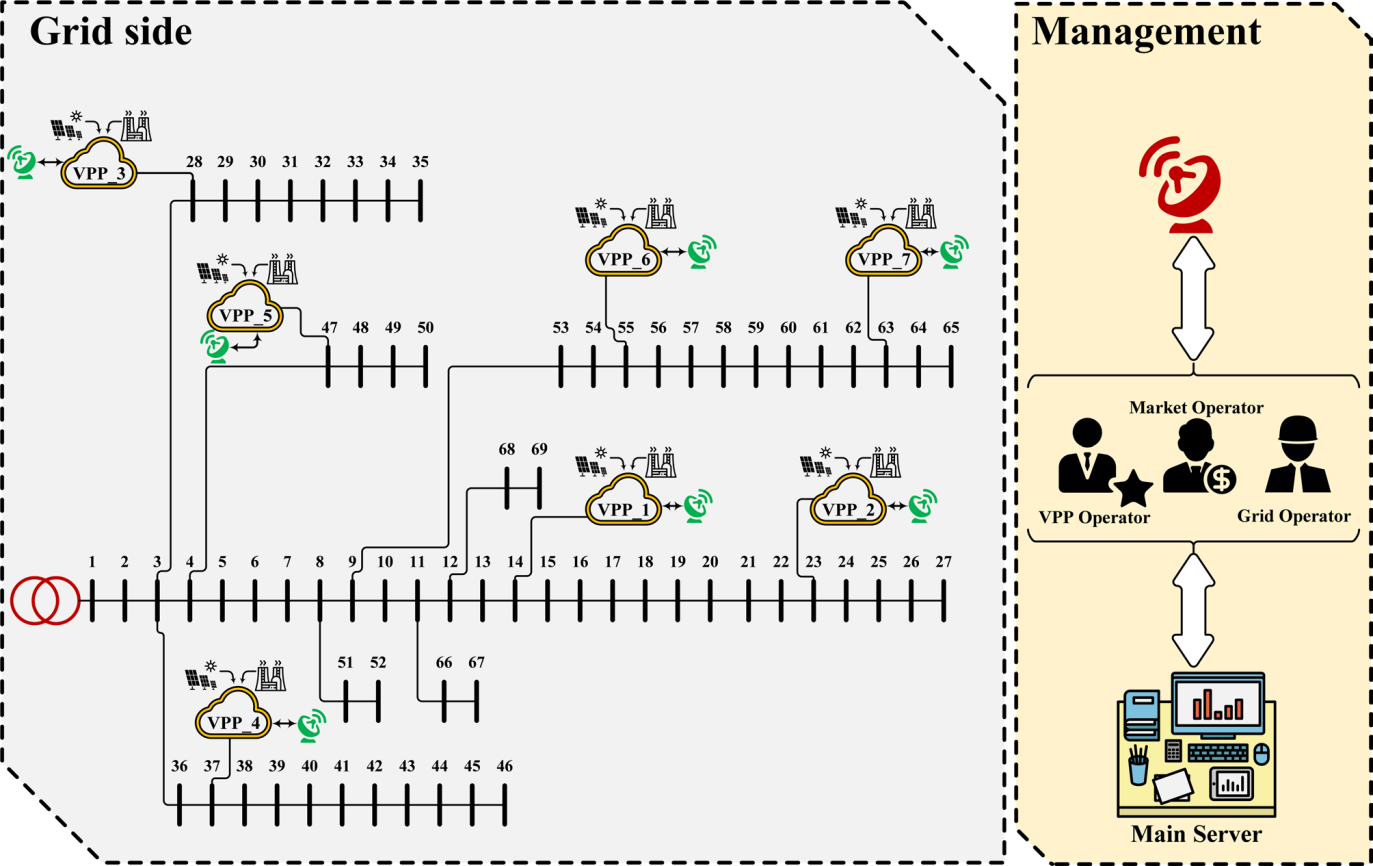
The 69-bus IDN in the presence of FRVPPs^11^.

**Fig. 4:**
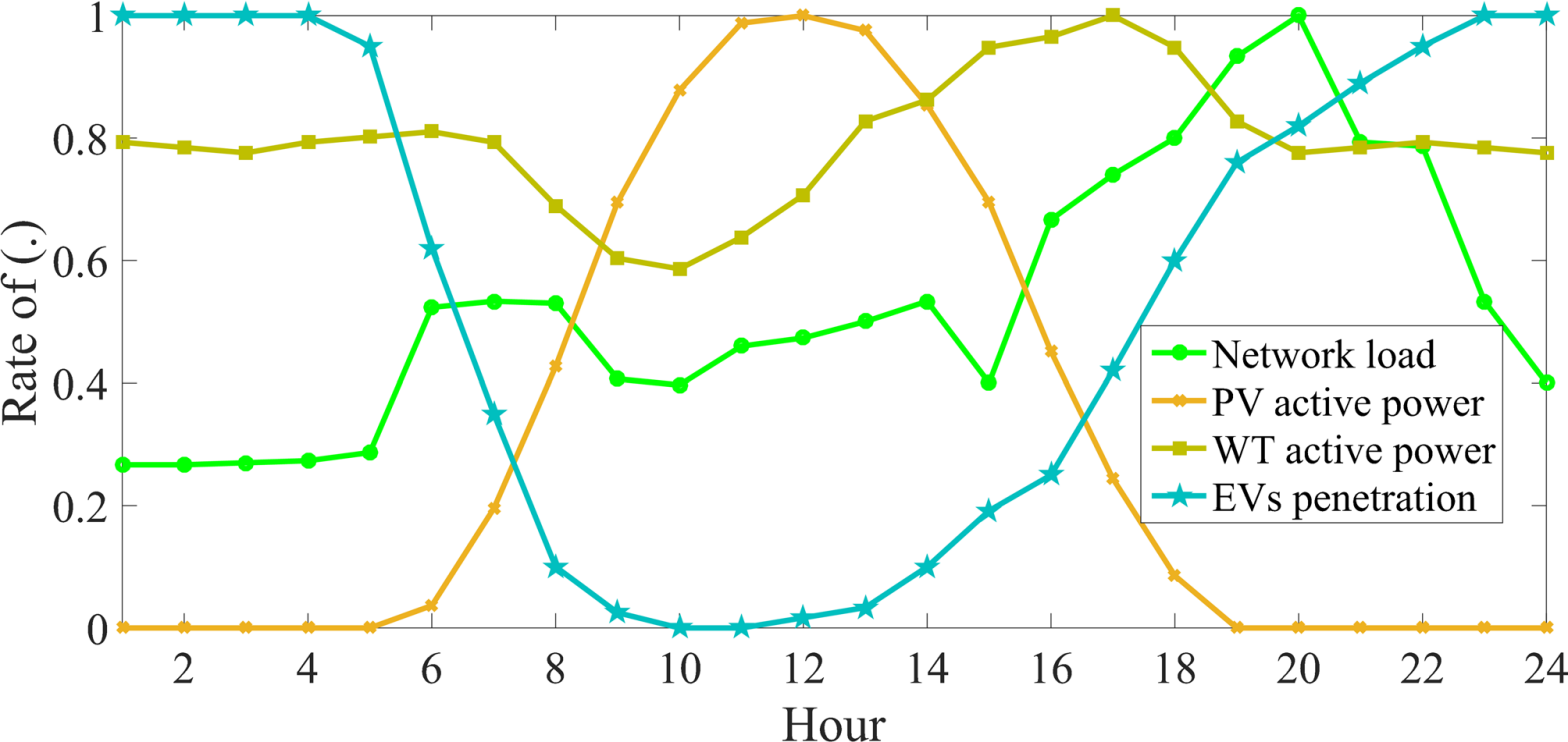
The daily curve of RESs power rate, load factor, and EVs penetration rate^1,11^.

In general, the proposed scheme with the mathematical model in Sects. 2 and 3 is capable of being implemented on various data of the power distribution network, resources, and storage devices. The scheme proposed in this section is applied to an IEEE 69-bus radial IDN^11^ as shown in Fig. 3. This network has 1 MVA base power and 12.66 kV base voltage. The allowable voltage range for non-critical (critical) loads is [0.9, 1.05] ([0.98, 1.02]) per unit (p.u.)^10,76–80^. The locations of critical loads in this network are buses 30–32, 49, 8–9, and 41–44. Other buses have non-critical loads. Characteristics of distribution lines and substations, along with peak load data, are extracted from^11^. The daily load profile is equal to the product of the peak load and the daily load factor curve (Fig. 4)^1,81–84^. The price of energy for the three off-peak (1:00–7:00), medium load (8:00–16:00 and 23:00–00:00), and peak (17:00–22:00) periods is 16 $/MWh, 24 $/MWh, and 30 $/MWh, respectively^1^. *K*_*Q*_ is assumed to be 8% based on^6^. Flexibility price in the upward mode for the periods (1:00–10:00 and 19:00–24:00), (11:00–12:00 and 17:00–18:00), and (13:00–16:00) is 37 $/MWh, 0.5 $/MWh, and 57 $/MWh, respectively^32^. This price in the downward mode is 95% of that in the upward mode. The network has renewable sources such as wind turbines (WTs) and photovoltaics (PVs). WTs with a capacity of 0.5 MW are installed at buses 17 and 49, and PVs with a capacity of 0.5 MW are installed at buses 41 and 53. Their daily power curve is equal to the product of their capacity and their generating power rate on a daily basis (Fig. 4)^11^. There are up to 7 flexible-renewable VPPs (FRVPPs) in the network. These FRVPPs contain renewable sources as well as flexibility sources (storage and responsive loads). The locations of FRVPPs are shown in Fig. 3. These FRVPPs are connected to the network by ES, forming a CVE. The loads on the connecting buses of CVEs are assumed to be controllable, so the ZIP load is used on these buses. In the ZIP load model, the coefficients *Z*, *I*, and *P* have values ​​of 0.3, 0.3, and 0.4, respectively^10^. Loss coefficients of ES, i.e., *α* and *β*, are 0.09 and 0.075, respectively^6^. Each ES has a capacity of 5 MVA. The active peak load per FRVPP is 200 kW. Each FRVPP has WT, PV, batteries, EVs, and responsive loads. The capacity of WTs and PVs in FRVPPs 1, 2, 6, and 7 is equal to 0.3 MW, but in other FRVPPs, it is equal to 0.5 MW. FRVPPs 1, 2, 6, and 7 each have up to 150 EVs, but other FRVPPs each have up to 200 EVs. Total EVs per FRVPP and the daily EV penetration rate are multiplied to calculate EVs per moment (Fig. 4)^6^. Other characteristics of EVs, such as charge/discharge rate, charge/discharge efficiency, battery capacity, initial energy, and minimum energy storage in the battery, are stated in^1,6]and [19^. Consumers at each FRVPP participate in DRP at a rate of 50%. There is a battery with a capacity of 1 MWh per FRVPP, where the minimum energy stored in it and its initial energy are 10% of the battery capacity. The charging and discharging rate of the battery is 0.5 MW, and the charging and discharging efficiency is 92%.

### Results and discussion

The proposed scheme is simulated in accordance with the data in Subsection 4.1 in the GMAS optimization software environment^31^. For the linear model (31)-(32), a regular 90-gon approximates the circular plane. Also, in the conventional piecewise linearization technique, five linear pieces are used. The uncertainty deviation is then assumed to be $\tilde {u}=r.BU$, where *r* represents the level of uncertainty.

A) *Evaluation of the convergence status of the proposed scheme*: Table 2 reports the convergence status of the deterministic problem in three nonlinear (1)-(18), linear (31)-(32), and BD-based linear models (36)-(53) for different solvers considering BD = 0. In the nonlinear model, IPOPT, CONOPT, LGO, and MINOS solvers^31^ are used to solve the problem. Among these algorithms, LGO cannot achieve the optimal solution. Other algorithms do not have a unique optimal solution, so IPOPT has a better situation than other solvers because it has a more optimal point (maximum F) and higher convergence speed (lower convergence iteration (CI) and lower computational time (CT)) than other algorithms. The CPLEX, CBC, and OSL algorithms are used to solve the linear problem^31^. A unique solution was obtained by these algorithms. The difference is in CI and CT, which makes CPLEX more suitable because its convergence speed is the highest (minimum CI and CT). Therefore, the CPLEX is used to solve the BD algorithm-based linear problem. According to Table 2, it is observed that with decreasing the convergence tolerance of BD (*ε*), the computational difference between the linear model and the linear formulation based on BD (difference between F values in these two models) is reduced significantly so that at *ε* = 0.01, the optimal solution is the same in the two models. From the perspective of computational time, the nonlinear IPOPT-based model has a CT of 923.5 s. However, in the CPLEX-based linear model, it has been reduced to about 21.4 s. That is, CT in the linear model is reduced by about 97.7% compared to the nonlinear model. The BD algorithm could greatly reduce the computational time so that at *ε* = 0.01, it has a CT of 13.1 s. This CT is reduced by about 38.8% (98.6%) compared to CT in the linear (nonlinear) model. Therefore, the BD algorithm is in a good position compared to other models because it has a unique optimal solution and considerably shorter computational time.

Table 3 presents the convergence status of problems (54)–(57), i.e., the problem of IDN operation considering the participation of CVEs in energy and active and reactive ancillary services markets, in the robust model for BD = 1. In comparison to a deterministic model (*r* = 0), the uncertainty level increases with increasing uncertainty level (r)., A measure of the objective function’s value (F_1_) decreases, and CI and computational time increase because, in the worst-case scenario based on^29^, the solution space is reduced. The optimal solution in CI and CT is higher than that of the deterministic model. The worst-case scenario has the worst scenario of uncertainties (discussed in Subsection 4.2. B)^29^ (i.e., the situation that causes the worst objective function compared to the deterministic model). The value of the objective function is less than that of the deterministic model. Finally, Table 4 reports the computational error of the power and voltage variables in the BD-based linear model compared to the main formulation based on the nonlinear model. Accordingly, the computational error of power is about 2.3%, but it is about 0.3–0.45% for voltage. Since the computational time in the BD method is very low, which corresponds to the operation goals in the operation problems, the computational errors in this regard can be ignored.

**Table 2:** Convergence status of the scheme obtained by different solvers in the deterministic model.

Model	Solver	F ($)	CI	CT (s)	Model status
Non-linear	**IPOPT**	**992.7**	**356**	**923.5**	**Feasible**
	CONOPT	948.5	410	1106	Feasible
	LGO	-			Infeasible
	MINOS	896.2	463	1365	Feasible
Linear	**CPLEX**	**1047.3**	**43**	**21.4**	**Feasible**
	CBC	1047.3	56	25.9	Feasible
	OSL	1047.3	85	28.6	Feasible
BD algorithm in robust model with BD = 0	CPLEX (ε = 0.2)	1047.6	41^#^/6^*^	10.4	Feasible
	CPLEX (ε = 0.1)	1047.0	42^#^/8^*^	11.9	Feasible
	**CPLEX (ε = 0.01)**	**1047.3**	**42** ^**#**^ **/11** ^*^	**13.1**	**Feasible**

^#^Convergence iteration for the CPLEX solver, *Convergence iteration of the BD algorithm.

**Table 3:** BD convergence status in the robust model with BD = 1.

Model	Solver	*r* = 0.1				*r* = 0.2			
		F_1_ ($)	CI	CT (s)	Model status	F_1_ ($)	CI	CT (s)	Model status
BD algorithm	CPLEX (ε = 0.2)	924.8	44/8^*^	12.1	Feasible	849.4	44^#^/9^*^	13.9	Feasible
	CPLEX (ε = 0.1)	925.4	45/10^*^	13.7	Feasible	849.9	45^#^/12^*^	15.5	Feasible
	**CPLEX (ε = 0.01)**	**925.7**	**45/14** ^*^	**14.8**	**Feasible**	**850.2**	**45** ^**#**^ **/16** ^*^	**16.7**	**Feasible**

^#^Convergence iteration for the CPLEX solver, *Convergence iteration of the BD algorithm.

**Table 4:** Calculation error of different variables in the BD-based linear model versus the original non-linear model in the deterministic model.

Model	BD-based linear model (BU = 0)	Non-linear	Calculation error (%)
Solver	CPLEX	IPOPT	
*P*_*DS*_ at 20:00 (p.u.)	2.078	2.126	2.26
*Q*_*DS*_ at 20:00 (p.u.)	1.291	1.322	2.34
Mean value of *V* (p.u.)	0.959	0.956	0.31
Mean value of *σ* (p.u.)	− 0.0454	− 0.0452	0.44

B) *Analyzing the worst-case uncertainty values*: Based on different values of BU and r, Table 5 presents the true values of uncertain parameters in the worst-case scenario. Accordingly, compared to the deterministic model scenario, it is evident that as BD and r increase (with BU = 0 or *r* = 0), the active and reactive load and the maximum energy consumption of EVs increase, but other uncertainties decrease. In other words, in the worst-case scenario, passive load and EV increase compared to the deterministic model. The increase in the load of EVs is associated with the increase in *E*^*up, u*^ and the decrease in *E*^*lo, u*^, *E*^*in, u*^, *CR*^*u*^, and *DR*^*u*^. By increasing *E*^*up, u*^ and decreasing *E*^*lo, u*^ and *E*^*in, u*^, the operation horizon of EVs will require more energy consumption. Reduced *CR*^*u*^ and *DR*^*u*^ cause EVs to demand power from VPP for longer hours. However, compared to the deterministic model, the worst-case scenario reduces the capacity of renewable energy production and energy prices. These cases lead to a decrease in the profit of CVEs in the worst-case scenario compared to the deterministic model based on Table 2.

**Table 5:** Uncertain parameter values in worst-case scenarios.

Parameter	BU = 0	BU = 0.5			BU = 1		
	*r* = [0 ∞)	*r* = 0	*r* = 0.1	*r* = 0.2	*r* = 0	*r* = 0.1	*r* = 0.2
$$\sum\nolimits_{{b,\tau }} {P_{{L\,b,\tau }}^{u}}$$ (p.u.)	57.8	57.8	60.7	63.6	57.8	63.6	68.6
$$\sum\nolimits_{{b,\tau }} {Q_{{L\,b,\tau }}^{u}}$$ (p.u.)	41.04	41.04	43.1	45.15	41.04	45.15	48.7
$$\sum\nolimits_{{b,\tau }} {P_{{PV\,b,\tau }}^{u}}$$ (p.u.)	20.32	20.32	19.3	18.3	20.32	18.3	16.8
$$\sum\nolimits_{{b,\tau }} {P_{{WT\,b,\tau }}^{u}}$$ (p.u.)	51.5	51.5	48.9	46.4	51.5	46.4	42.1
$$\sum\nolimits_{{b,\tau }} {CR_{{b,\tau }}^{u}}$$ (p.u.)	3.6	3.6	3.42	3.24	3.6	3.24	2.96
$$\sum\nolimits_{{b,\tau }} {DR_{{b,\tau }}^{u}}$$ (p.u.)	3.6	3.6	3.42	3.24	3.6	3.24	0.96
$$\sum\nolimits_{{b,\tau }} {E_{{b,\tau }}^{{lo,u}}}$$ (p.u.)	1.44	1.44	1.37	1.3	1.44	1.3	1.19
$$\sum\nolimits_{{b,\tau }} {E_{{b,\tau }}^{{in,u}}}$$ (p.u.)	1.44	1.44	1.37	1.3	1.44	1.3	1.19
$$\sum\nolimits_{{b,\tau }} {E_{{b,\tau }}^{{up,u}}}$$ (p.u.)	14.4	14.4	15.1	15.8	14.4	15.8	16.9
$$\sum\nolimits_{\tau } {\rho _{{E\,\tau }}^{u}}$$ ($/MWh)	550	550	528	495	550	495	457

**Fig. 5:**
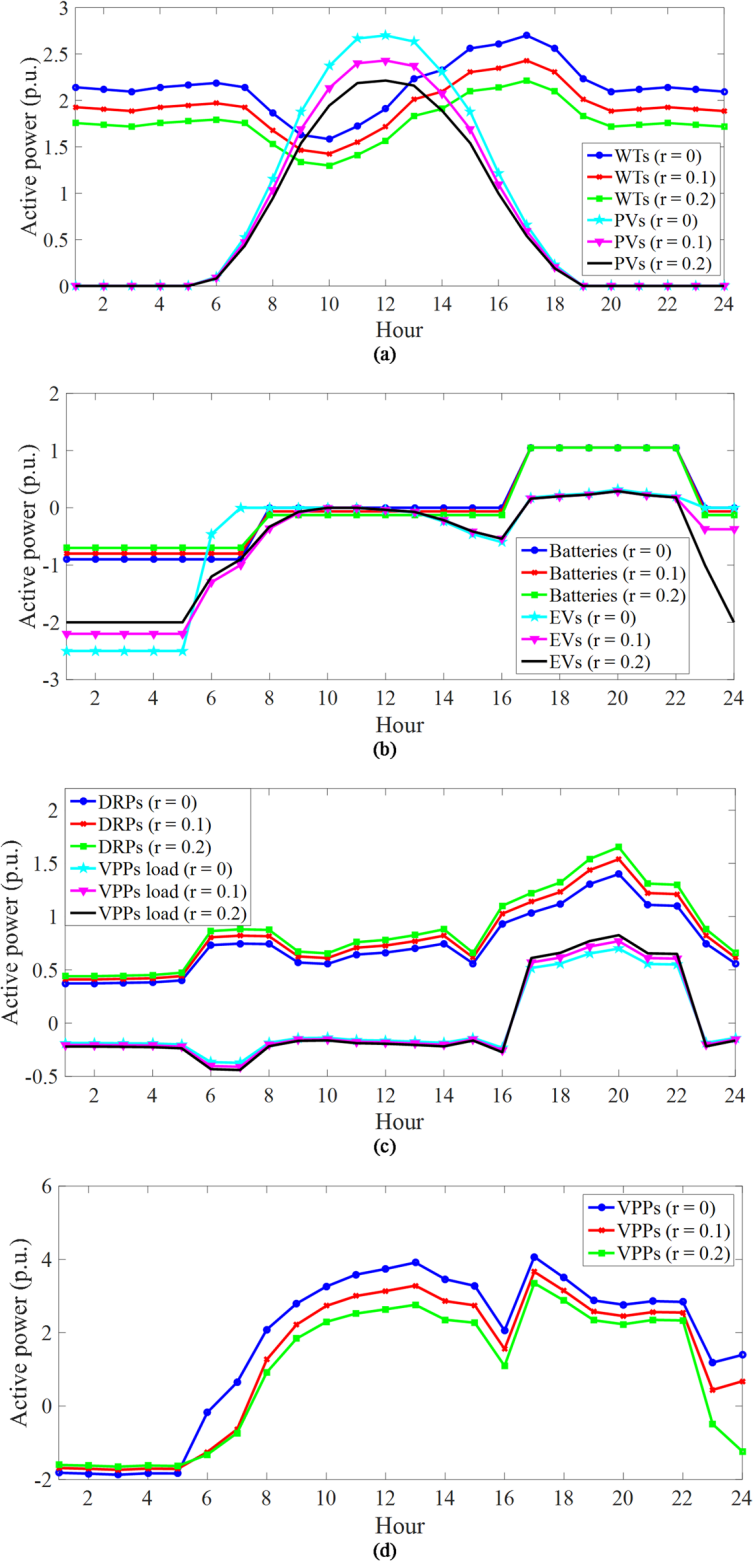
Daily active power curve of (**a**) RESs, (**b**) storages, (**c**) VPPs load and DRP, and (**d**) VPPs at different uncertainty levels for BU = 1.

C) *Evaluation of the performance of VPPs in CVEs*: Fig. 5 depicts the daily curves of the total active power and elements of VPPs for different uncertainty levels. Based on Fig. 5a, the daily active power curve of WTs and PVs is the same as the daily power rate curve of their output in Fig. 4. This is related to the fact that, according to Subsection 4.1, renewable sources generate active power when their capacity and generation power are multiplied. As another point, increasing the uncertainty level decreases the active power of these renewable sources in all operation hours compared to the deterministic model (*r* = 0). This figure confirms the results presented in Table 4 regarding the lower generation power capacity of renewable sources in the worst-case scenario.

The daily curve of the active power of storage devices, i.e., stationary (batteries) and mobile (EVs) storage, and the responsive loads are shown in Fig. 5b, c, respectively. Based on these figures, it can be seen that storage and responsive loads operate generally at low energy price hours (1:00–16:00 and 23:00–00:00), corresponding to the off-peak and medium load intervals in the charge mode or in the mode of receiving energy from VPPs. However, in hours of high energy prices (17:00–22:00) corresponding to the peak load period, they operate in the mode of discharging or injecting energy into VPPs. In Fig. 5b, EVs perform two charging operations – one to supply the energy they need to travel, which has a high energy level and appears between 1:00–7:00 and 23:00–00:00, and the other is when they are charged from 12:00–16:00 until they inject the stored energy of the EV batteries into VPPs or IDNs during the peak load period. This performance trend of storage devices and responsive loads aims to gain high financial profit in the energy market. For example, in the deterministic model (*r* = 0), batteries purchase 6.3 MWh (7 h × 0.9 MW according to Fig. 5b) energy at 16 $/MWh during 1:00–7:00 from IDN. Nonetheless, they sell the same amount of energy to IDN during peak hours at 30 $/MWh. The operation of these batteries is expected to increase the financial benefit of CVEs in the energy market. Note that as *r* increases, the charge interval of the storage devices increases. In batteries and responsive loads, increasing this range increases the efficiency of CVEs in enhancing IDN flexibility. However, in EVs, the increase in this range is due to the increase in *E*^*up, u*^ and the decrease in *E*^*lo, u*^, *E*^*in, u*^, *CR*^*u*^, and *DR*^*u*^, as well as the need for EVs to work together in CVE to enhance IDN flexibility. It is noteworthy that in the flexibility discussion, as given in (54)-(57), in deterministic models, power swings from renewable sources are minimized in the worst-case scenario, while CVEs minimize them in the worst-case scenario. According to Fig. 5b, since renewable sources have active generation capacity at all hours, CVEs are expected to operate at all hours for flexibility. The flexibility sources in CVEs, such as storage devices and responsive loads, are operating at most hours. Finally, it can be seen in Fig. 5b, c that as *r* increases, the discharge capacity of the battery does not change (because the battery injects energy equal to its maximum capacity to VPPs during peak hours), the discharge capacity of EVs decreases (because *DR*^*u*^ according to Table 4 decreases in the worst-case scenario), and the discharge capacity of responsive loads increases (because it depends on the active power of the load based on (11), and active power increases according to Table 4; Fig. 5c in the worst-case scenario).

Figure 5d displays the daily active power curve of VPPs, which depends on the performance of renewables, storage devices, responsive loads, and passive load based on (10). Finally, using this figure, during off-peak hours of 1:00–7:00, VPPs consume more energy because, at these hours, storage devices and responsive loads are in the charging mode according to Fig. 5b, c. However, at other hours, VPPs appear as power generators in the IDN. With increasing *r*, power consumption and generation of VPPs decrease. According to Fig. 5b, the decrease in power consumption is due to the decrease in power consumption of storage devices with increasing *r* during 1:00–7:00. The decrease in generation power is due to the decrease in generation power of power sources and storage devices with increasing *r* based on Fig. 5a, b.

**Fig. 6:**
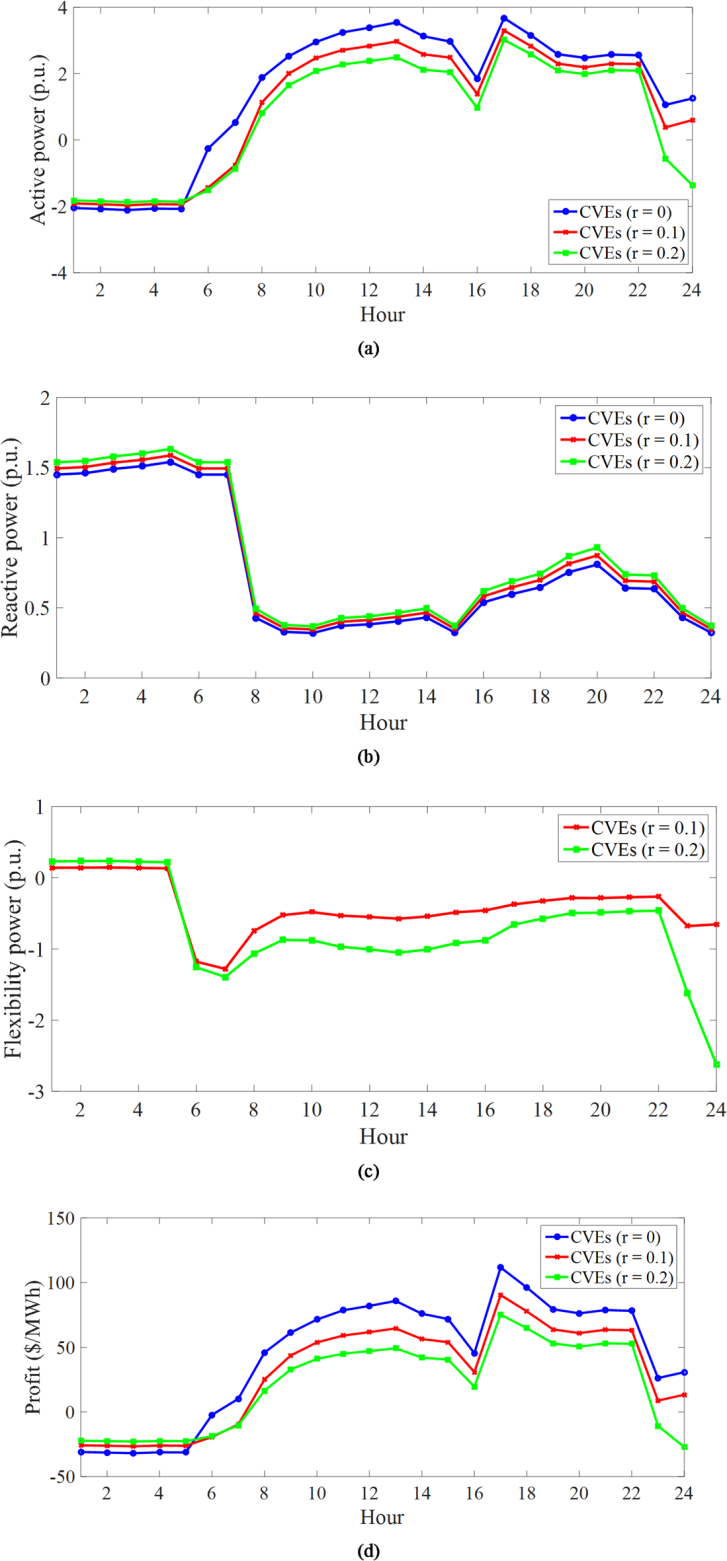
The daily curve of (**a**) active power, (**b**) reactive power, (**c**) flexibility power, and (**d**) profit of CVEs at different uncertainty levels for BU = 1.

**Fig. 7:**
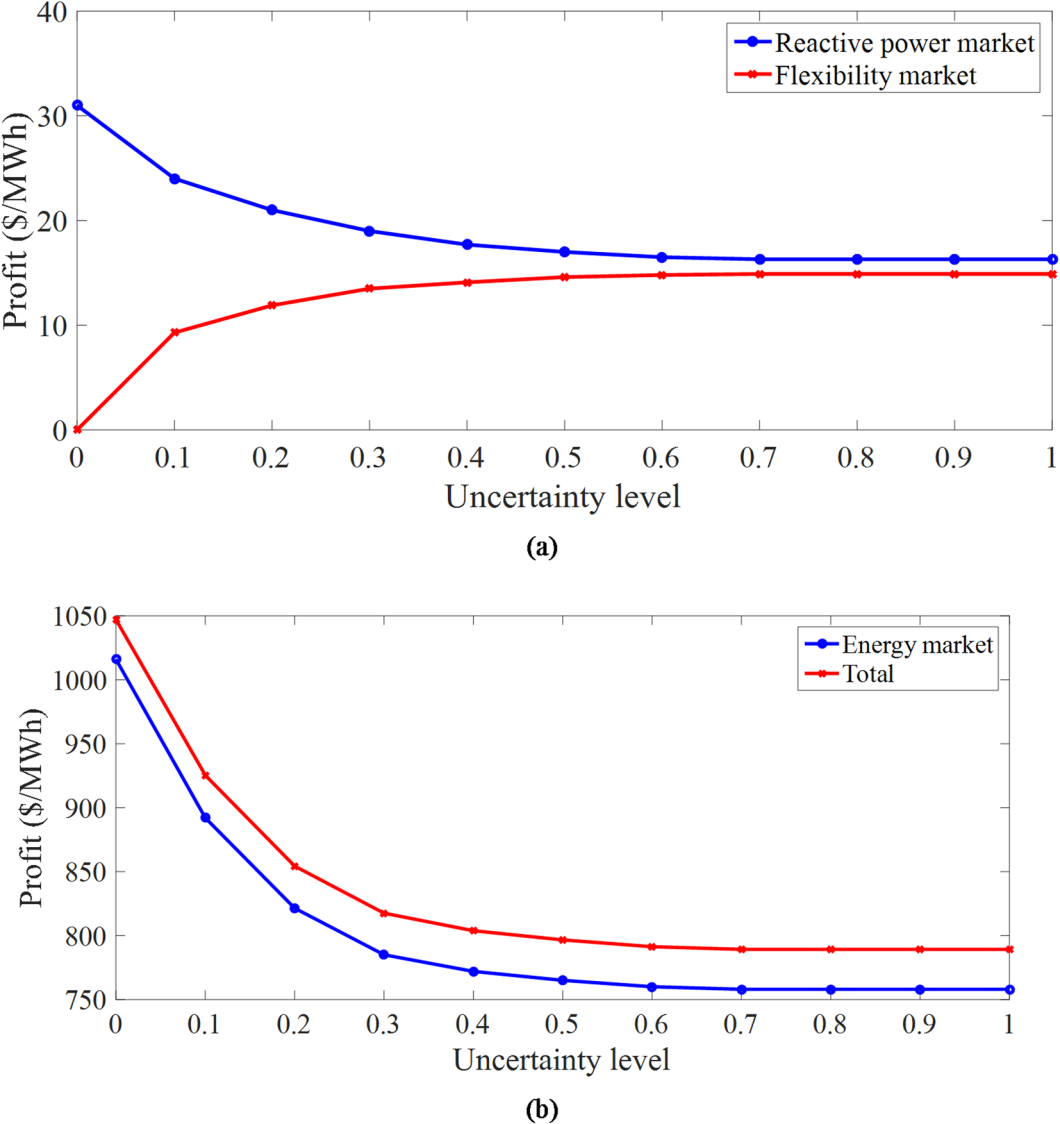
The curve of profit in uncertainty level at (**a**) reactive power and flexibility markets and (**b**) energy and all markets in BU = 1.

D) *Operational assessment*,* flexibility*,* and status of the economy of CVEs in IDN*: Fig. 6 displays the daily curve of power and flexibility of CVEs along with CVEs’ profit in energy, reactive power, and flexibility markets for BU = 1 for different uncertainty levels. The graph in Fig. 6a shows the daily active power curve of CVEs, the trend of which is the same as that of VPPs’ active power in Fig. 5b because, according to (16), VPPs and ES losses combine to form CVEs’ active power. Since the coefficients *α* and *β* are ​​less than 0.1 according to Subsection 4.1, the effect of the active power of VPPs is much greater than the effect of ES losses on the active power of CVEs. Changes in CVE active power will follow the trend of changes in VPP active power. CVEs are shown in Fig. 6b as a graph of their daily reactive power. Figure 7 illustrates that the maximum reactive power of CVEs injected into IDN appears during 1:00–7:00 because VPPs are in consumer mode at this time and receive significant active power from IDN. Therefore, to prevent high voltage drop, CVEs insert high-level reactive power to IDN during these hours. At other times, CVEs insert high active power to IDN as shown in Fig. 6a. To prevent overvoltage in IDN, CVEs inject low reactive power into IDN during these hours. CVE reactive power increases in peak load intervals during these hours due to the increase in the daily load factor curve shown in Fig. 4. Then, with increasing *r*, the amount of reactive power of CVEs injected into IDN increases in comparison to the deterministic model (*r* = 0) to prevent voltage drop due to the increased load in these conditions according to Table 4. The daily flexibility curve of CVEs is displayed in Fig. 6c. This power, based on (55), is equal to the difference between the active power of CVEs in the worst-case situation (*r* = 0.1 or *r* = 0.2), in contrast to a deterministic scenario (*r* = 0). It does not exist in the deterministic model based on Fig. 6c. A positive (negative) value of this power in this curve means that the CVEs are operating in an upward (downward) mode. CVEs operate in the upward mode during off-peak hours, 1:00–7:00, because in the worst-case scenario, active power consumption is lower during these hours than in the deterministic model based on Fig. 6a. Nonetheless, at other hours, they are in the downward mode, because at these hours, the generation power of CVEs in the worst-case scenario is the lowest versus the deterministic model based on Fig. 6a. Finally, as *r* increases, the flexibility of CVEs increases in both upward and downward modes. According to Fig. 5a, the active power generation of renewable sources differs significantly and increases compared to the deterministic model.

According to Fig. 6d, CVEs in the market of energy and ancillary services show a correlation between their daily profits and their active power, which is due to the very low reactive power price of flexibility compared to the energy price based on Subsection 4.1. This issue has influenced the profits of CVEs in the energy market more than in the ancillary services market.

This issue can be seen in Fig. 6. In other words, to maximize the profit of VPPs in this market based on the energy price signal, energy market encourages VPPs to supply the energy consumption of EVs and the energy required for charging batteries and DRPs in cheap energy price hours such as 1:00–7:00. It encourages VPPs to inject their maximum active power into the network during other hours when energy prices are high. VPPs inject maximum active power into the network as far as the network limitations allow. In the reactive power market, VPPs or ESs are encouraged to inject their maximum reactive power into the grid. VPPs inject high reactive power into the network based on the capacity limitation of the ES and network constraints.

Figure 7 depicts the profit curve of CVEs in various markets mentioned in terms of the uncertainty level for BU = 1. It is observed from Fig. 7a that with increasing *r*, the profit of CVEs in the reactive power market decreases. Although the reactive power of CVEs increases with increasing *r* according to Fig. 6b, the price of reactive power, which depends on the price of energy, decreases because the energy price, according to Table 4, decreases with increasing *r*. Now, the decrease in the profit of CVEs in the reactive power market by increasing *r* means that the effect of reducing the price of reactive power in the mentioned conditions is greater than that of increasing the reactive power of CVEs in these situations. Figure 7a shows that an increase in *r* increases the profitability of CVEs in the flexibility market. The reason is that, according to Fig. 6c, the flexibility has increased in the mentioned conditions. Nevertheless, the opposite is true for the profits of CVEs in the energy market based on Fig. 7b. With increasing *r*, the energy price/active power of CVEs decreases according to Table 4; Fig. 6a, so increasing *r* leads to a decrease in CVE profits in the energy market. Finally, based on Fig. 7b, it is observed that with increasing the uncertainty level, the total profit of CVEs in the mentioned markets decreases.

(E) *Assessment of IDN operation status*: Table 5 shows energy losses, maximum voltage drop, and maximum overvoltage values in buses without and with critical loads for BU = 1 for the following case studies:

- Case I: Power flow studies.
- Case II: Proposed scheme.

The energy loss for the network is equal to the sum of the energy generated by the distribution substation, resources, and CVEs (in generator mode) minus the sum of energy consumed by loads and CVEs (in consumer mode). If this issue temporarily disrupts the energy balance of the network, the power loss resulting from this imbalance will be calculated. According to the results in Table 6, it is observed that by managing the power of CVEs and voltage regulation in IDN, compared to power flow studies, the proposed scheme improved operation indices. In the worst-case scenario (*r* = 0.2), it reduced energy losses by about 16.3% ((2.299–1.925)/2.299) versus Case I. Also, according to the proposed scheme, establishing a maximum overvoltage of 0.011 p.u. (lower than the allowable value of 0.05 (0.02)) in buses with non-critical (critical) loads has maximized voltage drop in buses with non-critical loads by about 47.8% for *r* = 0.2 compared to Case I. It reduced the maximum voltage drop across the buses with critical load to the permissible level of 0.02 p.u. compared to about 0.088 p.u. for Case I.

**Table 6:** Different cases of operation indices at BU = 1.

Index	Case I	Case II		
		*r* = 0	*r* = 0.1	*r* = 0.2
Energy loss (MWh)	2.299	1.582	1.768	1.925
Maximum voltage drop (p.u.) in IDN	0.092	0.053	0.050	0.048
Maximum over-voltage (p.u.) in IDN	0	0.011	0.009	0.008
Maximum voltage drop (p.u.) in buses including critical load	0.088	0.02	0.02	0.02
Maximum over-voltage (p.u.) in buses including critical load	0	0.005	0.003	0.002

## Conclusion

The paper presented an operation problem for an IDN in the presence of a CVE system. CVEs participate in energy, reactive power, and flexibility markets in which VPP is in an aggregating format for power sources, storage devices, and a responding load. The deterministic schematic model of the proposed system is responsible for maximizing the profit of CVEs in the energy and reactive power markets by observing the AC-OPF limitations and the operation model of CVEs. An approximate linear model was then developed to derive this scheme. Various factors, including ARO, energy prices, renewable power, and mobile storage device energy consumption, were used to model energy uncertainty and load uncertainty. The formulation of CVE participation in the flexibility market was further added to the proposed scheme. In addition to the constraints of the robust model and the flexibility model of CVEs, since its objective function is the total profit of CVEs in the mentioned markets, it includes the constraints of both. Next, the optimal solution was determined using the BD algorithm. Finally, numerical results showed that the algorithm could extract a unique optimal solution. The calculation time was much lower than that of the proposed scheme’s linear and nonlinear models, and the computational error of 2.3% and 0.4% for power and voltage in this algorithm, respectively, compared to the original nonlinear model of the proposed scheme, can be ignored with respect to the computational time. Then, with the optimal operation of power sources, responsive loads, and storage in the form of VPP, CVEs were consumers (producers) of energy in off-peak (medium-peak) load periods. CVEs generated high reactive power during the off-peak period compared to other hours to prevent voltage drop due to the energy consumption of CVEs during this interval. They were generally in upward (downward) flexibility mode during the off-peak (medium-peak) load periods. Ultimately, this performance of CVEs led them to receive financial benefits in most operating hours of these markets. Furthermore, when worst-case scenarios occurred, the energy consumption (production) increased (decreased) compared to the deterministic model. However, in the worst-case scenario with an uncertainty level of 0.2, the proposed scheme reduced energy losses and maximum voltage drop in buses with non-critical loads by 17% and 48% versus power flow studies, respectively. It increased the maximum voltage drop across buses with critical load to the permissible value of 0.02 p.u. whereas in power flow studies, it had a value greater than 0.02 p.u.

The DC/AC converter used in VPP for the proposed design can play an effective role in compensating for the existing harmonics in the power distribution network. However, this capability of VPPs was not included in this scheme, so future researchers are recommended to focus on the proposed plan considering the harmonic model of the power distribution network and VPP.

## Data Availability

The datasets used and/or analysed during the current study available from the corresponding author on reasonable request.

## Abbreviations

ARO: Adaptive robust optimization
AC-OPF: AC optimal power flow
AC-PF: AC power flow
BD: Benders decomposition
BURO: Bounded uncertainty-based robust optimization
CI: Convergence iteration
CT: Computational time
CVE: Coupling of the virtual power plant and electric inverter
DA: Day-ahead
DSO: Distribution system operator
DRP: Demand response program
ES: Electric spring
EV: Electric vehicle
FR: Flexibility resource
FRVPP: Flexi-renewable virtual power plant
HSRO: Hybrid stochastic/robust optimization
IDN: Intelligent distribution system
IGBT: Insulated-gate bipolar transistor
LAC-OPF: Linearized AC optimal power flow
MG: Microgrid
MP: Master problem
NLP: Nonlinear programming
PDF: Probability distribution function
PV: Photovoltaic
SP: Sub-problem
RES: Renewable energy sources
RFEMS: Risk-averse flexi-intelligent energy management system
RT: Real-time
UT: Unscented transformation
VPP: Virtual power plant
ZIP: Constant impedance, current, and power

## Constants

*A*: CVEs and incidence buses matrix (the element *i,b* is 1 if the *i*th CVE is connected to bus *b*; otherwise, it is 0.)
*B*: Bus and distribution line occurrence matrix (the element *b,k* is 1 if the distribution line is between buses b and *k*; otherwise, it is 0.)
*b*_*DL*_*, g*_*DL*_: Susceptibility and conductivity of distribution lines (p.u.)
*C*: Space vector of CVE in the network (element *b* of the vector is 1 if CVE is connected to bus *b*; otherwise, is 0.)
*CR, DR*: Charging and discharging rates of electrical vehicles in storage (p.u.)
*E*^*lo*^*, E*^*in*^*, E*^*up*^: Maximum stored energy, minimum stored energy, and initial stored energy (p.u.)
*K*_*Q*_: The ratio between prices of reactive power and energy
*P*_*L*_*, Q*_*L*_: Loads power (p.u.)
*P*_*PV*_*, P*_*WT*_: PV and wind power (p.u.)
$$S_{DL}^{up} ,S_{DS}^{up} ,S_{CVE}^{up}$$: Power max on distribution lines, distribution substations, and CVEs (p.u.)
*sl *: Line slope in the piecewise linearization technique
*V*^*LF*^: Voltage amplitude in power flow studies (p.u.)
*V*^*lo*^*, V*^*up*^: Minimum and maximum amplitude of voltage (p.u.)
*Z, I, P*: Constant parameters in constant impedance, current, and power load model (ZIP)
*α**, **β*: Loss coefficients of electric inverter or spring (ES) in p.u.
*η*^*CH*^*, **η*^*DCH*^: Charge and discharge efficiency of the storage
*φ*_*U*_*, **φ*_*L*_: Flexibility price in upward and downward modes ($/MWh)
*ρ*_*E*_: Energy price ($/MWh)
*ψ*: Demand response program (DRP)
*Δδ*: Angle deviation (rad)

## Control variables

*CR*^*u*^*, DR*^*u*^: Uncertain variable of *CR* and *DR* (p.u.)
*E*^*lo,u*^*, E*^*in,u*^*, E*^*up,u*^: Uncertain variable of *E*^*lo*^, *E*^*in*^, and *E*^*up*^ (p.u.)
*P*_*CH*_*, P*_*DCH*_: Charge-discharge power (p.u.)
*Q*_*CVE*_: Reactive power of CVE (p.u.)
*P*_*DR*_: DRP active power (p.u.)
$$P_{L}^{u} ,Q_{L}^{u}$$: Uncertain variables *P*_*L*_ and *Q*_*L*_
$$P_{PV}^{u} ,Q_{WT}^{u}$$: Uncertain variables *P*_*PV*_ and *Q*_*WT*_ (p.u.)
$$\rho_{E}^{u}$$: Uncertain variable of *ρ*_*E*_ ($/MWh)

## Dependent variables

*F*: Amount of profits generated by CVEs in energy and reactive power markets ($)
*F*_*1*_: CVE profits in the energy, reactive power, and flexibility markets ($)
*F*_*L*_*, F*_*U*_: Flexibility power in downward and upward modes (p.u.)
*G*: Dual function of *F*
*P*_*CVE*_: Active power of CVE (p.u.)
$$P_{CVE}^{ + } ,P_{CVE}^{ - }$$: Positive and negative components of *P*_*CVE*_ (p.u.)
*P*_*VPP*_: VPP active power (p.u.)
*P*_*DL*_*, Q*_*DL*_: Active and reactive power of the distribution line (p.u.)
*P*_*DS*_*, Q*_*DS*_: Active and reactive power of the distribution substation (p.u.)
*P*_*LO*_: Active power loss of ES (p.u.)
*V, **Δ**V*: Voltage amplitude and voltage deviation (p.u.)
*λ**, **μ*: Dual variables
*σ*: Voltage angle (rad)

## Indices

*b *: Bus
*i *: CVE
*j *: Line segment in the piecewise linearization technique
*k *: Bus
*τ*: Operation hour
*ω*: Sides of a regular polygon
